# In Vivo Interactions of Nucleic Acid Nanostructures With Cells

**DOI:** 10.1002/adma.202314232

**Published:** 2024-09-12

**Authors:** Yu Xiao, Zhihui Liang, Moldir Shyngys, Aiana Baekova, Suen Cheung, Mathias Billy Muljadi, Qianqian Bai, Lula Zeng, Chung Hang Jonathan Choi

**Affiliations:** ^1^ Department of Biomedical Engineering The Chinese University of Hong Kong Shatin New Territories Hong Kong; ^2^ Center for Neuromusculoskeletal Restorative Medicine Hong Kong Science Park Shatin New Territories Hong Kong

**Keywords:** bio‐nano interaction, gene delivery, nanomedicines, nucleic acid nanotechnology, preclinical research

## Abstract

Nucleic acid nanostructures, derived from the assembly of nucleic acid building blocks (e.g., plasmids and oligonucleotides), are important intracellular carriers of therapeutic cargoes widely utilized in preclinical nanomedicine applications, yet their clinical translation remains scarce. In the era of “translational nucleic acid nanotechnology”, a deeper mechanistic understanding of the interactions of nucleic acid nanostructures with cells in vivo will guide the development of more efficacious nanomedicines. This review showcases the recent progress in dissecting the in vivo interactions of four key types of nucleic acid nanostructures (i.e., tile‐based, origami, spherical nucleic acid, and nucleic acid nanogel) with cells in rodents over the past five years. Emphasis lies on the cellular‐level distribution of nucleic acid nanostructures in various organs and tissues and the cellular responses induced by their cellular entry. Next, in the spirit of preclinical translation, this review features the latest interactions of nucleic acid nanostructures with cells in large animals and humans. Finally, the review offers directions for studying the interactions of nucleic acid nanostructures with cells from both materials and biology perspectives and concludes with some regulatory updates.

## Graphical abstract

Nucleic acid nanostructures have been intracellular drug carriers for nanomedicine applications for two decades, but their interaction with cells in vivo has gained clarity only recently. This review features their cellular‐level distribution in various organs and the cellular responses induced in rodents, large animals, and humans. It concludes with directions for probing cell‐nanoparticle interactions and regulatory updates for clinical translation.

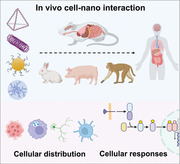

## 1 Introduction

Advances in nucleic acid nanotechnology since the 1980s have supported the assembly of nucleic acid building blocks (e.g., plasmids and oligonucleotides) into nucleic acid nanostructures with structural programmability (based on Watson‐Crick base pairs and secondary structure recognition interactions) and spatial addressability (chemical modification at specific sites of the nanostructure).^[^ ^1^ ^]^ The original inspiration for nucleic acid nanotechnology was to take DNA, a central biomolecule for storing and transmitting genetic information, “out of its biological context” for controlling molecular self‐assembly at the nanometer length scale.^[^ ^2^ ^]^ Thus, the classical applications of nucleic acid nanostructures were mostly far from biomedical, say molecular machines,^[^ ^3^ ^]^ logical circuits,^[^ ^4^ ^]^ and lattice engineering.^[^ ^5^ ^]^ Intriguingly, two decades later, nucleic acid nanotechnology began to embrace the biological roots of DNA when it became apparent that nucleic acid nanostructures do not induce severe toxicity or immunogenicity,^[^ ^6^ ^]^ can carry biological cargoes,^[^ ^7^ ^]^ enter mammalian cells,^[^ ^8^ ^]^ and show enhanced resistance against nuclease degradation.^[^ ^9^ ^]^

That nucleic acid nanostructures did not require the aid of cationic transfection agents for cellular entry was a striking revelation for three reasons. To start with, electrostatic repulsion between polyanionic nucleic acid nanostructures and cell membranes (also negatively charged) does not necessarily prohibit cellular entry.^[^ ^10^ ^]^ Large gene cargoes, such as DNA plasmid and messenger RNA (mRNA), do not readily enter cells due to electrostatic repulsion. Consequently, nanomedicine researchers developed liposomes,^[^ ^11^ ^]^ lipid nanoparticles (LNPs),^[^ ^12^ ^]^ and polymeric nanoparticles (NPs)^[^ ^13^ ^]^ with cationic groups for electrostatically complexing the gene cargoes and achieving intracellular delivery, but cationic carriers may induce cytotoxicity and immunogenicity. Extracellular vesicles^[^ ^14^ ^]^ cross the cell membrane by endocytosis or hydrophobic interaction, but their production with defined physicochemical parameters (size and shape) and subsequent purification (especially when scaled up) are challenging. Inorganic NPs^[^ ^15^ ^]^ do not necessitate cationic groups for cellular entry either, but their long‐term retention, biotransformation, and toxicity in vivo remain unclear. Regarding the second reason, 3D nucleic acid nanostructures enter cells abundantly, but their constituent unassembled linear (1D) oligonucleotides do not;^[^ ^16^ ^]^ this result underscores the role of nanoscale architectural control of DNA building blocks on cell‐NP interaction. Finally, the pronounced cellular entry of nucleic acid nanostructures empowers them to deliver various cargo types for nanomedicine applications, including fluorescent dyes for cellular imaging,^[^ ^17^ ^]^ small molecule drugs for killing cancer cells,^[^ ^18^ ^]^ and nucleic acids for regulating biological or disease pathways (e.g., antisense,^[^ ^19^ ^]^ small interference RNA (siRNA),^[^ ^20^ ^]^ small hairpin RNA (shRNA),^[^ ^21^ ^]^ and microRNA (miRNA)^[^ ^22^ ^]^) that may ultimately lead to disease treatment. As these nucleic acids have a short blood circulation half‐life, low resistance against serum nucleases, and no inherent ability to be delivered to specific tissues and cells,^[^ ^23^, ^24^ ^]^ hybridizing nucleic acids to or using them to constitute the final nucleic acid nanostructure has emerged as a promising strategy for cellular gene delivery. Summing up, over the past decade, nucleic acid nanotechnology as a field has acknowledged the importance of “translation” to stay relevant and engender an enduring impact,^[^ ^25^ ^]^ as evidenced by the plethora of studies on new nanostructure design and therapeutic efficacy evaluation using rodent disease models. Despite this encouraging trajectory toward translation, clinical use of nucleic acid nanostructures has yet to come to fruition with the most advanced clinical trials now in Phase 0/1. A critical bottleneck may stem from our inadequate understanding of how nucleic acid nanostructures interact with cells at the diseased site, say their distribution among different cell types of an organ (e.g., liver^[^ ^26^ ^]^ and lung^[^ ^27^ ^]^) and the biological responses induced by their cellular entry (e.g., inhibition of disease pathways^[^ ^28^ ^]^ and signaling kinases^[^ ^29^ ^]^). Rigorous studies on in vivo cell‐NP interactions should inspire more efficacious applications of nucleic acid nanostructures.

**Figure 1:**
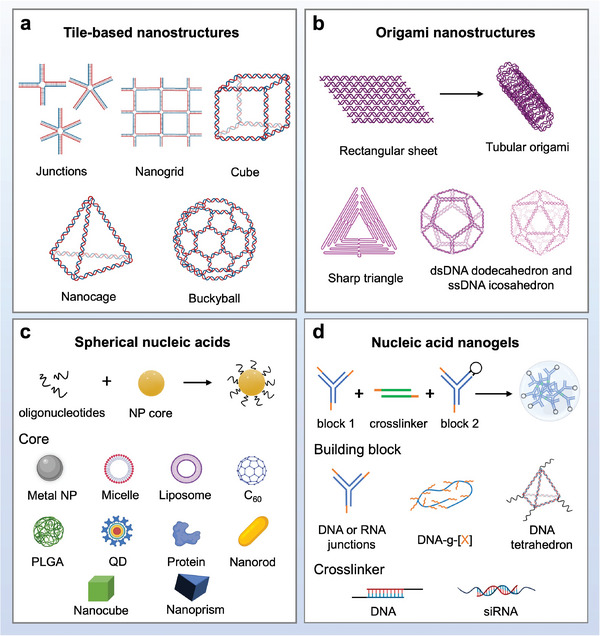
Schematic illustration of four representative types of nucleic acid nanostructures, including a) tile‐based, b) origami, c) spherical nucleic acids, and d) nucleic acid nanogels. PLGA: poly (lactic‐co‐glycolic acid); QD: quantum dot. DNA‐g‐[X]: DNA grafted polymers or drug molecules. Created with BioRender.com.

In this review, we summarize the recent progress in dissecting the in vivo cell‐NP interactions of four representative types of nucleic acid nanostructures, including tile‐based structures, origami‐based structures, spherical nucleic acids, and nucleic acid nanogels (**Figure** **1**). This work represents a significant advance from our past review of the cell‐NP interactions of nucleic acid nanostructures in vitro in 2019;^[^ ^30^ ^]^ at that point, we focused on the biological pathways of cellular entry and intracellular trafficking in vitro given the dearth of comprehensive studies on cell‐NP interactions in vivo. Here, we capture the growth of the field since then by i) covering the in vivo cellular‐level distribution of administered nucleic acid nanostructures and the cellular responses induced upon their cellular entry in different organs and tissue types in rodents, ii) featuring cell‐NP interactions in large animals and humans, and iii) offering directions for mechanistic studies and translation. This work is distinct from yet complementary to other contemporary reviews on the structural design of nucleic acid nanostructures for gene therapy,^[^ ^31^ ^]^ applications for cancer treatment and tissue engineering,^[^ ^32^ ^]^ various types of cargoes loaded,^[^ ^33^ ^]^ and methods for characterizing and overcoming the delivery barriers to the tumor and lymph node.^[^ ^34^ ^]^ This review serves to showcase the in vivo interaction of nucleic acid nanostructures with cells rather than sheer biomedical applications. Despite the plethora of biomedical applications of nucleic acid nanostructures, our basic knowledge of their cell‐NP interaction remains weak and the published experimental data often fail to address if a given nucleic acid nanostructure reached the desired cell type after injection into the body. It is our earnest wish that researchers could build more useful and effective targeting nucleic acid nanostructures by considering these interactions, thereby accelerating clinical translation.

## 2 Classical Nanomedicine Applications of Nucleic Acid Nanostructures

### 2.1 Tile‐based Nanostructures

In 1982, Seeman conceived the assembly of tile‐based DNA nanostructures using three‐arm or four‐arm junctions with sticky ends as building blocks. His vision was to manipulate the sequences of DNA oligonucleotides and design their sequences to create immobile and semi‐mobile junctions for building stable nanostructures.^[^ ^35^ ^]^ After developing DNA branched junctions with 3–4 arms^[^ ^36^ ^]^ and 5–6 arms,^[^ ^37^ ^]^ Seeman reported the DNA nanocube in 1991^[^ ^38^ ^]^ and followed up with octahedrons^[^ ^39^ ^]^ and 2D nanocrystal.^[^ ^40^ ^]^ The field then expanded with the introduction of other shapes, such as tetrahedron^[^ ^41^ ^]^ and bipyramid^[^ ^42^ ^]^ by Turberfield, nanoribbon and nanogrid^[^ ^43^ ^]^ by LaBean, multiple prism hybrid structures^[^ ^44^, ^45^ ^]^ by Sleiman, and polyhedra (e.g., icosahedron,^[^ ^46^ ^]^ tetrahedron, dodecahedron, and buckyball^[^ ^47^ ^]^) and complex shapes (e.g., kleetopes of polyhedra^[^ ^48^ ^]^) by Mao. Similarly, RNA junctions serve as building blocks for assembling nanoscale hexamers^[^ ^49^ ^]^ and tile‐based structures^[^ ^50^ ^]^ (e.g., tectosquare,^[^ ^51^ ^]^ dodecahedron,^[^ ^52^ ^]^ and nanocage^[^ ^53^ ^]^). Tile‐based nanostructure typically requires single‐stranded DNA (ssDNA) oligonucleotides with 3–4 different sequences for construction, therefore its production complexity and assembly error are low^[^ ^54^ ^]^ and its assembly yield is high (>80%).^[^ ^55^ ^]^ These are all attractive traits for large‐scale production required for large‐animal and clinical tests. For nanomedicine applications, the most commonly used tile‐based nanostructure is the tetrahedral framework nucleic acid (tFNA). Besides its established ability to enter cells and carry drugs and gene cargoes, it is also surprisingly a self‐therapeutic agent for multiple diseases; no additional chemical or biological drugs are needed.^[^ ^56^, ^57^ ^]^ However, for tile‐based nanostructures, it is challenging to determine the optimal location of “crossovers” that enable the bridging of two double DNA helices by a pair of shared ssDNA oligonucleotides. A non‐optimal crossover location may lead to structural defects and a lower formation yield.^[^ ^58^ ^]^

Keum et al. constructed a DNA pyramid with an antisense DNA loop to inhibit the green fluorescent protein in vitro.^[^ ^59^ ^]^ Kim et al. attached fluorescent dyes to a DNA tetrahedron and loaded doxorubicin (DOX), an anti‐cancer small molecule drug, via intercalation for killing cancer cells in vitro.^[^ ^60^ ^]^ Lee et al. built a DNA tetrahedron with oligonucleotides containing a nick complementary to the overhang of siRNA strands against the firefly luciferase reporter gene; intravenous (i.v.) administration of this tetrahedron enabled tumor‐targeted delivery and gene silencing in mice.^[^ ^61^ ^]^ Mou et al. replaced thymidine with floxuridine, a nucleoside analog of thymidine and an anti‐cancer drug, when constructing a DNA polyhedron; i.v. delivery of this polyhedron inhibited tumor growth.^[^ ^62^ ^]^ Kim et al. loaded DNA pyramids with DOX via intercalation and bound streptavidin‐conjugated caspase‐3 (an apoptotic protein) to the constituent biotinylated DNA strands; their i.v. delivery reduced tumor size.^[^ ^63^ ^]^ Tian et al. constructed a DNA tetrahedron with one edge containing a nucleolin‐responsive aptamer sequence and loaded drug molecules internally. Upon binding to nucleolin on tumor cells, the nucleolin‐responsive edge dehybridizes to trigger drug release.^[^ ^64^ ^]^

To boost the performance of tile‐based nanostructures, one may introduce chemical modifications, say using a phosphorothioate (PS) backbone to prevent nuclease degradation of nanocages,^[^ ^63^ ^]^ choosing uniform sticky ends to reduce self‐assembly errors of the tile‐based building block,^[^ ^65^ ^]^ attaching cationic lipids to improve stability and cellular interaction of tetrahedra,^[^ ^66^ ^]^ using special junctions to construct ultrastable nanostructures,^[^ ^67^ ^]^ adding specific ions to improve the yield of assembling polyhedra,^[^ ^68^ ^]^ and attaching aptamers to improve delivery of tetrahedra to target tissues.^[^ ^69^ ^]^

### 2.2 Origami

In 2006, Rothemund introduced DNA origami nanostructures, in analogy to the art of paper folding.^[^ ^70^ ^]^ He used hundreds of short DNA oligonucleotides (50–60 bases long), or staple strands, to fold a long ssDNA scaffold (typically 7000–8000 bases long) via DNA hybridization. The staple strands pinch together parts of the scaffold, form a designated arrangement of helices, and hence fold the scaffold into a 2D or 3D nanostructure of a specific shape or pattern. This discovery spurred the development of DNA origami nanostructures with complex curvature^[^ ^71^ ^]^ and multi‐arm junction vertices^[^ ^72^ ^]^ as well as inspired the automated design of nonregular or oblique shapes.^[^ ^73^ ^]^ In 2014, Geary et al. reported an RNA origami by folding a single RNA strand into 2D RNA origami nanosheets with alternating stacks of tertiary kissing loop motifs and double crossover junctions; the 2D nanosheets can be arranged into hexagonal lattices.^[^ ^74^ ^]^ The folding process does not require heating, an attractive feature for large‐scale production. Also, origami synthesis can leverage computer modeling to design suitable clipping strands and yield origami nanostructures with a broad range of sizes, shapes (straight vs curved), and dimensions (2D vs 3D). The well‐defined and highly addressable origami nanostructure allows for the integration of drug molecules and targeting ligands with controllable numbers and position,^[^ ^75^, ^76^ ^]^ thus opening the door for investigating the interaction between nanostructures and the cell membrane or receptors. However, the one‐pot reaction of DNA origami self‐assembly often creates many by‐products, such as dimers, trimers, and other aggregates.^[^ ^77^ ^]^ Of the hundreds of DNA strands added to the reaction, only <20% will be incorporated into the final product.^[^ ^55^ ^]^ Therefore, post‐assembly annealing is crucial for high‐quality purification, but this step is sensitive to reaction duration, volume, dilution, and purification methods [e.g., polyethylene glycol (PEG) precipitation and gel filtration].^[^ ^78^ ^]^

Geary et al. developed the RNA Origami Automated Design software for kilobase‐sized nanostructures by optimizing sequences and identifying folding barriers.^[^ ^79^ ^]^ Automation can also support post‐synthesis purification of origami nanostructures. Chau et al. added the crude product of DNA origami nanostructures to a mixture of carboxylated magnetic microparticles, salt, and PEG, causing the selective Ψ‐condensation^[^ ^80^ ^]^ of larger origamis. Magnetic concentration of the microparticle‐bound origamis removed the staple strands, and pipetting by a liquid handling robot led to large‐scale purification of the origamis from microparticles.^[^ ^81^ ^]^ Jiang et al. used DNA origami nanotriangles for encapsulating DOX via intercalation to kill chemoresistant cancer cells.^[^ ^82^ ^]^ The authors later conjugated the tumor‐suppressing gene p53 to the DOX‐encapsulated DNA nanotriangle for inhibiting tumors.^[^ ^83^ ^]^ Schüller et al. reported a DNA origami nanotube with staple strands decorated with unmethylated cytosine‐phosphate‐guanine (CpG) DNA (an agonist of the Toll‐like receptor 9; TLR9)^[^ ^84^ ^]^ to improve delivery to primary splenic macrophages and enhance immune response. Pradhan et al. built an antiviral agent by conjugating multiple nanobodies against pseudorabies virus to the surface of a DNA origami icosahedron. This nanostructure binds to the virus particle and hence blocks the interaction between the virus and host cell in vitro.^[^ ^85^ ^]^ Recently, Yin et al. hybridized tissue plasminogen activator proteins (an anti‐thrombosis drug) to a DNA origami nanosheet, folded the sheet to form a nanotube, and fastened the nanotube with a DNA duplex comprised of a thrombin‐responsive aptamer and a complementary locking strand.^[^ ^86^ ^]^ This tube triggered thrombolysis in an ischemic stroke rat model and a pulmonary embolism mouse model. Zeng et al. developed an anti‐tumor vaccine based on a square‐block DNA origami hybridized with CpG strands as the adjuvant and ovalbumin (OVA) proteins as the antigen.^[^ ^75^ ^]^ Upon subcutaneous (s.c.) injection to tumor‐bearing mice, this origami accumulated in the nearest draining lymph nodes and activated dendritic cells, CD8 T^+^ cells, Th1‐polarized CD4^+^ cells, and natural killer cells.

Some challenges associated with origami nanostructures are cellular uptake, immunogenicity, and stability. Coating virus capsid proteins onto a DNA origami nanorectangle increased cellular entry.^[^ ^87^ ^]^ Coating with a PEG‐modified bilayer improved stability and bioavailability as well as lowered immune response.^[^ ^88^ ^]^ Exclusion of phage genes or CpG domains from the M13mp18 scaffold, the structural basis of most origami nanostructures that originates from bacteriophage genomic DNA, decreased immune response in vivo.^[^ ^89^, ^90^ ^]^ Choosing a compact, lattice‐based structure over an open, wireframe design facilitated cellular uptake of origami nanostructures via scavenger receptors.^[^ ^91^ ^]^ Finally, adding more global twists (or bases per helical turn) to a DNA origami nanotube of a given length enhanced the loading of DOX via intercalation and cytotoxicity against cancer cells.^[^ ^92^ ^]^

### 2.3 Spherical Nucleic Acids (SNAs)

In 1996, Mirkin pioneered SNAs, nucleic acid nanostructures containing an outer shell of oligonucleotides that are densely functionalized to the surface of an NP template core.^[^ ^93^ ^]^ As 3D nanostructures, SNAs differ from their structural cousins of linear oligonucleotide (1D) and circular plasmids (2D). Gold‐cored SNAs, derived from the dense attachment of thiolated oligonucleotides to the gold core via gold‐sulfur linkages, mark the first generation of SNAs and are now widely used for gene regulation,^[^ ^94^ ^]^ drug delivery,^[^ ^95^ ^]^ and molecular diagnostics.^[^ ^96^ ^]^ Later years have witnessed the expansion of the structural possibilities of SNAs that incorporate various sizes and types of i) NP (e.g., iron oxide,^[^ ^97^ ^]^ silica,^[^ ^98^ ^]^ micelle,^[^ ^99^ ^]^ protein,^[^ ^100^ ^]^ and bucky ball^[^ ^101^ ^]^) as the template core, ii) oligonucleotides (e.g., antisense DNA,^[^ ^102^ ^]^ aptamer,^[^ ^103^ ^]^ immunomodulatory DNA,^[^ ^104^ ^]^ miRNA,^[^ ^105^ ^]^ and siRNA^[^ ^106^ ^]^) with chemical modifications for stability against serum nuclease (e.g., locked nucleic acid,^[^ ^107^ ^]^ PS backbone,^[^ ^108^ ^]^ and peptide nucleic acid^[^ ^109^ ^]^) to constitute the outer shell, and iii) chemical linkages between the NP core and oligonucleotide (e.g., cyclic disulfide for silver NP,^[^ ^110^ ^]^ and tocopherol tails for liposome,^[^ ^111^ ^]^ and dibenzocyclooctyne for quantum dot coated with an azide‐containing polymer shell^[^ ^112^ ^]^). A critical advantage of SNA synthesis is its structural predictability. Given an appropriate conjugation chemistry for linking oligonucleotides to the NP core, the resultant SNA will attain its characteristic outer 3D oligonucleotide shell, often independent of oligonucleotide type, length, and sequence. Moreover, choosing an NP template core of a defined size and shape largely determines the final size and shape of the final SNA nanostructure. However, SNA synthesis can be time‐consuming due to the stepwise salt‐aging method used for screening the electrostatic repulsion between the multiple densely conjugated polyanionic oligonucleotides on the NP core.^[^ ^113^ ^]^

Of these structural parameters, the 3D outer shell of oligonucleotides most pronouncedly governs SNA's more favorable interaction with cells than single‐stranded oligonucleotides in vitro (e.g., enhanced stability against nuclease degradation,^[^ ^112^ ^]^ binding to Class A scavenger receptor that mediates cellular entry,^[^ ^11^ ^]^ gene regulation,^[^ ^113^ ^]^ and immunomodulation^[^ ^104^ ^]^) and in vivo (e.g., crossing biological barriers and cellular entry in the skin^[^ ^114^ ^]^ and brain^[^ ^115^ ^]^). To decipher cell‐nano interactions, one may employ NP cores that are fluorescent^[^ ^116^ ^]^ or magnetic^[^ ^117^ ^]^ for whole‐cell imaging, plasmonic for biosensing,^[^ ^118^ ^]^ metallic for quantifying cellular contents by inductively coupled plasma mass spectrometry (ICP‐MS), and imaging subcellular trafficking to organelles by transmission electron microscopy (TEM),^[^ ^11^ ^]^ and anisotropic for determining the directionality of NP movement.^[^ ^119^ ^]^

### 2.4 Nucleic Acid Nanogels

In the 2000s, researchers employed DNA junctions to construct 3D hydrogels with a stable crosslinked network; such hydrogels are structurally distinct from nucleic acid‐containing hydrogels in which nucleic acids are simply loaded as cargoes with no apparent role in the overall structure.^[^ ^120^ ^]^ Examples are hydrogels based on branched DNA molecules with sticky ends by Luo,^[^ ^121^ ^]^ pH‐responsive hydrogels derived from three‐arm junctions via i‐motif,^[^ ^122^ ^]^ and heat‐ or enzyme‐responsive hydrogels via hybridization of the sticky ends of three‐arm junctions by Liu.^[^ ^123^ ^]^ While these DNA hydrogels could load small molecules and oligonucleotides,^[^ ^124^ ^]^ their large size precluded effective cellular entry to interrogate biological responses or disease pathways. In 2015, Tan reported DNA nanohydrogels whose overall NP size is tunable (25–250 nm) by tuning the ratio of various types of three‐arm junction building blocks; when conjugated with DNAzyme strands against matrix metallopeptidase 9 (a marker of cell proliferation), the nanogel entered cancer cells and inhibited their growth in vitro.^[^ ^125^ ^]^ This work laid the experimental foundation for using nucleic acid nanogels as carriers of various therapeutic cargoes in nanomedicine applications, ranging from small molecules^[^ ^126^ ^]^ and peptides^[^ ^127^ ^]^ to nucleic acids (e.g., siRNA,^[^ ^128^ ^]^ miRNA,^[^ ^129^ ^]^ and mRNA^[^ ^130^ ^]^). Synthesis of nucleic acid nanogels entails either established building blocks commonly used for tile‐based nanostructures (L‐shape, Y‐shape, or tetrahedron)^[^ ^131^ ^]^ or simple DNA block copolymers as scaffolds.^[^ ^132^ ^]^ Changing the size, type, and amount of building blocks and scaffold allows for flexible tuning of the size, density, and strength of the nanogel network. Finally, the porous structure of nanogel empowers the facile loading of drug and dye cargoes. As the key limitation, most existing nanogels are relatively large (≈100 nm), so they have a limited ability to access different in vivo diseased sites due to their pronounced clearance by the liver and spleen. More compact nucleic acid nanogels are desirable for therapeutic applications.

Ding et al. designed therapeutic siRNA strands against the oncogene polo‐like kinase 1 (PLK1) with sticky ends to crosslink DNA‐grafted polycaprolactone brushes. The resultant DNA‐polymer nanogel buries siRNA strands in the core to protect them from nuclease degradation and exposes DNA strands to the periphery for cellular entry by mimicking the SNA architecture. Its i.v. injection led to gene silencing and tumor reduction.^[^ ^132^ ^]^ Guo et al. conjugated multiple hydrophobic photosensitizer molecules (pheophorbide A) to the PS modification sites on the backbone of four component DNA strands for assembly to a tetrahedron; similarly, addition of siRNA strands against programmed death ligand‐1, a checkpoint protein regulating immune evasion, as crosslinking strands led to the supramolecular assembly to form a nanogel for cancer photoimmunotherapy.^[^ ^128^ ^]^ Hu et al. designed three DNA building blocks with cytosine‐rich overhangs for self‐assembly to form a DNA nanogel; the building blocks were modified with CpG oligonucleotides, single‐stranded RNA (ssRNA) as TLR9 agonists or OVA peptides as antigen. Upon cellular entry, the low pH in the late endosome triggers the conversion to i‐motifs, disassembly of the nanogel for drug release, and ultimately an immune response in tumor‐bearing mice.^[^ ^133^ ^]^ Recently, Yan et al. constructed a DNA nanogel using siRNA duplexes against heat shock protein 70 (highly expressed in the tumor) to crosslink multiple tFNAs. They intercalated DOX molecules and coated the nanogel with polydopamine, an adhesive polymer^[^ ^134^ ^]^ with photothermal properties and robust entry to cells.^[^ ^135^ ^]^ This polydopamine‐DNA hybrid nanogel empowers synergistic chemo‐ and photothermal therapy to reduce tumor growth upon i.v. injection.^[^ ^136^ ^]^

## 3 Recent In Vivo Interactions of Nucleic Acid Nanostructures with Cells

From Sections 3.1 to 3.8, we present the in vivo cell‐NP interactions of nucleic acid nanostructures based on rodent studies in the past six years. The lowest animal order for testing the pharmacology and efficacy of NPs is rodent (mouse and rat). We include studies that offer direct in vivo evidence of the i) cellular‐level distribution of nucleic acid nanostructures in an organ or tissue or ii) cellular responses induced by their cellular entry. Any organ or tissue omitted below stems from the lack of reports with in vivo evidence based on the two criteria. These stringent selection criteria are critical to condense the focus of our review, reflect the current progress of the fields of nucleic acid nanostructures and bio‐nano interactions, and distinguish this review from other contemporary reviews. Based on these criteria, we could identify only 34 qualified reports over the past 5–6 years for inclusion from Sections 3.1 to 3.8. In Section 3.9, we present the emerging in vivo cell‐NP interactions in non‐mammalian species. Typical low‐order animals for proving the feasibility of imaging are zebrafish and worms.

### 3.1 Heart and Aorta

From the injection site of an i.v. injection, NPs are transported to the heart via the veins, and blood is delivered to the right ventricle.^[^ ^137^ ^]^ We review the cell‐NP interactions in the cardiovascular system. The vascular endothelium in normal tissue is dense and intact, difficult for NPs to cross the vascular wall and accumulate in the heart.^[^ ^138^ ^]^ We showed that SNAs, like other types of NPs,^[^ ^139^ ^]^ do not readily accumulate in the heart or aorta of healthy mice.^[^ ^140^ ^]^ Rather, NPs accumulate in blood vessels with atherosclerosis, a condition marked by chronic inflammation and remodeling of the vascular wall induced by hyperlipidemia. Accumulation of lipids, macrophages, and cholesterol in the endothelium causes the formation of atherosclerotic plaques that block blood flow, underpinning stroke and ischemic heart disease.

Previously, we reported the use of SNAs for naturally targeting atherosclerotic plaques, without using known targeting ligands of plaque cell types. In our proof‐of‐concept studies, we attached noncoding polythymidine DNA oligonucleotides to a PEG‐coated superparamagnetic iron oxide NP (PEG‐SPION) to construct a DNA‐based SNA (DNA‐SPION).^[^ ^140^ ^]^ Upon i.v. injection into apolipoprotein E knockout (ApoE^–/–^) mice fed on a high‐cholesterol diet, an established disease model of atherosclerosis, the DNA‐SPIONs accumulate in the aorta and aortic root (the primary sites of plaques) more rapidly and abundantly than PEG‐SPIONs. Notably, by flow cytometry analysis of the plaque cells, we detected that DNA‐SPIONs associate with aortic dendritic cells, endothelial cells (ECs), and M2 macrophages more preferentially than PEG‐SPIONs, proof of the 3D DNA shell in promoting entry to plaque cells.

**Figure 2:**
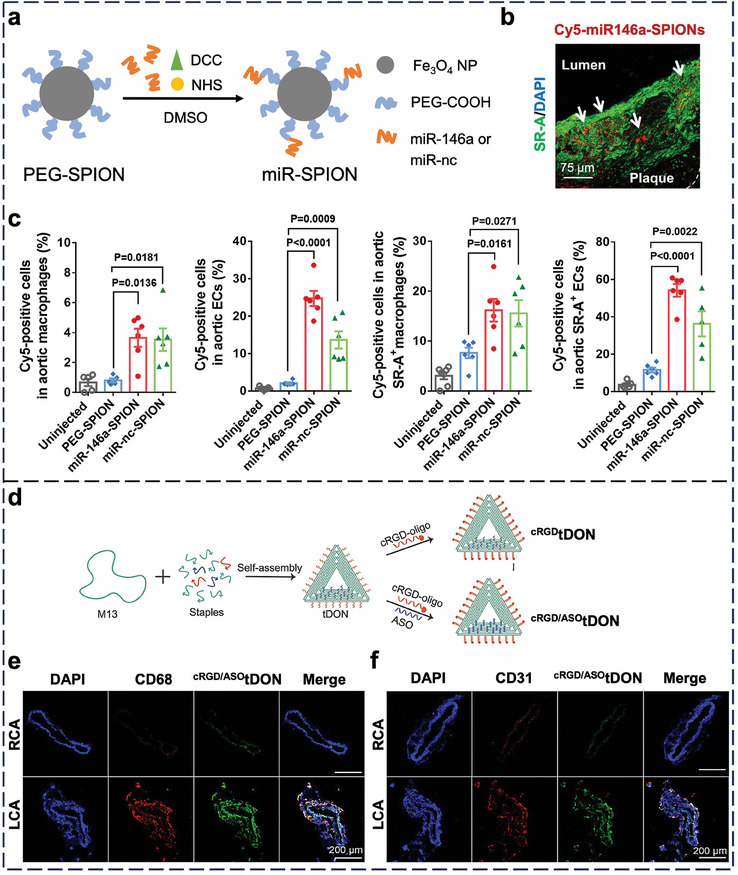
In vivo cell‐NP interaction of nucleic acid nanostructures in atherosclerotic plaques of the heart and aorta in ApoE^−/−^ mice. a) Synthesis of miR‐SPIONs. DCC: *N,N*′‐Dicyclohexylcarbodiimide; NHS: N‐hydroxysuccinimide; DMSO: dimethyl sulfoxide. PEG‐SPION functionalized with scrambled miRNA sequences (miR‐nc) is denoted as “miR‐nc‐SPION”. b) Representative confocal image of the aortic root confirmed the colocalization of Cy5‐labeled miR‐146a‐SPIONs (red) with aortic SR‐A‐rich macrophages or ECs (green) 2 h post‐injection. Blue = nuclei (stained by DAPI). c) Flow cytometry analysis of the cells in the aorta revealed stronger association of miR‐146a‐SPIONs (red) and miR‐nc‐SPIONs (green) to total aortic macrophages (first from left), total aortic ECs (second from left), SR‐A‐rich (SR‐A^+^) aortic macrophages (second from right), and SR‐A^+^ aortic ECs (first from right) than PEG‐SPIONs (blue). In addition, flow cytometry analysis revealed a stronger association of miR‐146a‐SPIONs (red) and miR‐nc‐SPIONs (green) to SR‐A^+^ aortic macrophages than total aortic macrophages, and a stronger association with SR‐A^+^ aortic ECs than total aortic ECs 2 h post‐injection. Reproduced with permission.^141^ Copyright 2022, National Academy of Sciences. d) Synthesis of anti‐miR‐33‐loaded and cRGD‐conjugated DNA origami (^cRGD/ASO^tDON). cRGD: cyclo‐RGDfK. Representative confocal images confirmed the colocalization of FITC‐labeled ^cRGD/ASO^tDON (green) with (e) macrophages (CD68; red) and (f) endothelial cells (CD31; red) in the left carotid artery (LCA) where atherosclerotic plaques exist rather than the right carotid artery (RCA) 7 h post‐injection. Blue = nuclei (stained by DAPI). Reproduced with permission.^[^ ^144^ ^]^ Copyright 2024, American Chemical Society.

Later, we investigated the molecular basis for the enhanced plaque delivery of SNAs. Here, we attached ≈275 PS‐modified miR‐146a strands to a PEG‐SPION to build a miR‐146a‐SPION of ≈70 nm in diameter (**Figure** **2**a).^[^ ^141^ ^]^ MiR‐146a negatively regulates the nuclear factor kappa‐light‐chain‐enhancer of activated B cells (NF‐ĸB) proinflammatory signaling pathway linked to atherogenesis. Like T_30_‐SPIONs, miR‐146a‐SPIONs naturally enter plaque cells upon i.v. injection (Figure 2b). Flow cytometry studies of plaque cells revealed 4‐6‐fold higher association of miR‐146a‐SPIONs to aortic macrophages and 10–15‐fold higher association to aortic ECs than PEG‐SPIONs. In turn, the superior entry of miR‐146a‐SPIONs into plaque cells led to their elevated accumulation in the plaque [1.2% of the injected dose (%ID); one of the highest delivery efficiencies in the field] over PEG‐SPIONs (0.4%ID). Critically, we used flow cytometry to prove that the miR‐146a‐SPIONs associated with aortic macrophages with enriched expression levels of SR‐A ≈5‐fold more likely than aortic total macrophages (which include both SR‐A‐rich and SR‐A‐deficient macrophages). Likewise, we obtained a similar preferential association of miR‐146a‐SPIONs to SR‐A‐rich aortic ECs than total aortic ECs (Figure 2c). These data provide the first in vivo evidence of SR‐A mediating the cellular entry of SNAs and imply that other types of nucleic acid‐based nanostructures that enter cells via SR‐A76^[^ ^142^ ^]^ are viable gene carriers for atherosclerosis. Finally, miR‐146a‐SPIONs reduced the plaque area in the aorta and aortic root, and the contents of macrophages and endothelial cells in the plaques, suggesting reduced plaque inflammation. However, the half‐life time of the miR‐146a‐SPION is relatively short (1.89 h), and improvement of blood circulation is desirable for reduced drug dosage. By establishing miRNA‐based SNA as a dual agent for plaque delivery and treatment, we invite the community to utilize nucleic acid nanostructures for regulating other genes or pathways linked to atherosclerosis,^[^ ^143^ ^]^ inflammation, or cardiovascular diseases in general by adjusting the oligonucleotide sequence. Recently, Ma et al. reported an anti‐atherosclerosis DNA origami nanostructure with three elements, 1) a cyclic pentapeptide ligand cRGD for targeting αvβ3 integrin receptors on atherosclerotic macrophages and ECs, 2) anti‐miR‐33 for promoting cholesterol removal and macrophage reprogramming, and 3) a triangular DNA origami as a dual scavenger of reactive oxygen species and a nanocarrier for cRGDs and anti‐miR‐33 (Figure 2d).^[^ ^144^ ^]^ Upon i.v. injection into ApoE^–/–^ mice with partially ligated carotid artery, this nanostructure preferentially accumulated in the plaque‐bearing left carotid artery over the normal right carotid artery and primarily entered atherosclerotic macrophages (Figure 2e) and ECs (Figure 2f) 7 h post‐injection.

Our understanding of how nucleic acid nanostructures interact with the heart and aorta is still in infancy. Delivery to the heart and aorta is inefficient (partially due to the small size of atherosclerotic plaques), with ≤1% ID reaching both sites. There is a dearth of quantitative calculation of in vivo distribution to various cell types (e.g., macrophages versus endothelial cells), making objective assessments of in vivo cellular targeting efficiency challenging.

### 3.2 Lung

**Figure 3:**
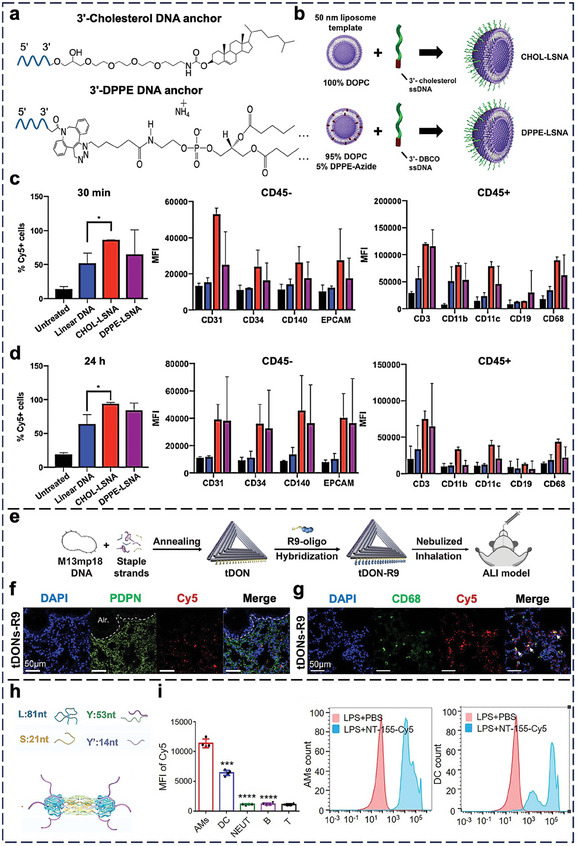
In vivo cell‐NP interaction of nucleic acid nanostructures in the lungs. a) Illustration of 3′‐Cholesterol DNA anchor and 3′‐1,2‐bis(diphenylphosphino)ethane (DPPE) DNA anchor. b) Preparation of CHOL‐LSNA and DPPE‐LSNA. DBCO: dibenzocyclooctyne. Flow cytometry analysis of total accumulation of Cy5‐DNA in immune (CD45^+^) and nonimmune cells (CD45^−^) from linear DNA and LSNAs in the lungs c) 30 min and d) 24 h post‐injection. CD3: marker of T cells; CD19: marker of B cells; CD11b: marker of neutrophils; CD11c: marker of dendritic cells; CD68: marker of macrophages; Epithelial cell adhesion molecule (EPCAM): marker of epithelial cells. MFI: mean fluorescence intensity. Statistical significance comparing percent Cy5^+^ cells was calculated by one‐way ANOVA with Tukey's post hoc test; *p < 0.05. Reproduced with permission.^[^ ^149^ ^]^ Copyright 2020, American Chemical Society. e) Preparation of peptide R9‐modified triangular DNA origami (tDON‐R9). f,g) Representative confocal images revealed the cellular‐level distribution of Cy5‐labeled tDON‐R9 (red) in the lipopolysaccharide‐challenged lung tissue 2 h after intratracheal administration. PDPN: podoplanin, a marker of type I alveolar epithelial cells. CD68: marker of alveolar macrophage. White arrows indicate the colocalization of CD68 (green) and tDONs‐R9 (red). Blue = nuclei (stained by DAPI). Reproduced with permission.^[^ ^156^ ^]^ Copyright 2024, American Chemical Society. h) Preparation of DNA nanotube with anti‐miR‐155 (NT‐155). i) Immune cell distribution of NT‐155 in bronchoalveolar lavage fluid after intratracheal administration. Data presented as mean value ± SD (*n =* 4). ***p < 0.001, ****p < 0.0001 relative to the control group AM (alveolar macrophage). Reproduced with permission.^[^ ^155^ ^]^ Copyright 2023, Elsevier.

After leaving the heart, i.v. injected NPs pass through the pulmonary circulation,^[^ ^137^ ^]^ so we next review the interactions of nucleic acid nanostructures in the lungs. NPs must diffuse through the mucosal layer (with a pore size of 20–200 nm) to the underneath periciliary layer before clearance by the mucociliary activity. Past studies mostly utilized nucleic acid nanostructures for suppressing lung cancer^[^ ^145^, ^146^, ^147^ ^]^ or inflammation,^[^ ^148^ ^]^ but even in healthy animals, our understanding of how nucleic acid nanostructures interact with various lung cell types in vivo remains scarce.^[^ ^20^ ^]^ Ferrer et al. showed that the affinity of DNA oligonucleotides attached to a liposome core determines the distribution of SNAs in the lungs upon i.v. injection (**Figure** **3**a,b). Surprisingly, liposomal SNAs (LSNAs) modified with low‐affinity cholesterol tails (CHOL‐LSNAs) for DNA conjugation accumulate in the lungs more abundantly than LSNAs modified with high‐affinity diacylglycerol lipid tails (DPPE‐LSNAs).^[^ ^149^ ^]^ The CHOL‐LSNAs initially associate with nonimmune cells (epithelial cells, ECs, fibroblasts, and blood‐derived stem cells), but they later associate with immune cells (Figure 3c,d).

The distribution of nucleic acid nanostructures in healthy and diseased animals can differ. Huang et al. designed DNA nanotetrahedra consisting of drug and gene cargoes for treating acute lung injury (ALI), induced by the inflammation of the air‐blood barrier (or alveolar‐capillary barrier) in the lung.^[^ ^150^ ^]^ Spermidine, the drug cargo, not only mediates the assembly of DNA tetrahedron with its positive charge, but also exhibits anti‐inflammatory, antioxidant, and antiaging properties.^[^ ^151^ ^]^ For the gene cargo, siRNA inhibits the mTOR pathway related to macrophage autophagy, a key anti‐inflammatory process during ALI.^[^ ^152^, ^153^, ^154^ ^]^ Upon i.v. injection, the DNA tetrahedra accumulates in the lungs of ALI mice ≈3 times more pronouncedly than in the lungs of healthy mice. Loss of capillary endothelium/alveolar epithelium integrity during inflammation may promote passive accumulation of nanostructures. While the authors did not address their distribution among various lung cell types, they showed that the DNA nanotetrahedra led to M1 to M2 macrophage polarization in alveoli, inhibition of pro‐inflammatory cytokines, and recovery of ventilation function.

I.v. administration for lung delivery can be inefficient because it suffers from first‐pass elimination by the liver and degradation by blood nucleases.^[^ ^155^ ^]^ Intratracheal administration for direct delivery to the lungs through the respiratory tract is an emerging attractive alternative. Wang et al. prepared cell‐penetrating R9 peptide‐modified triangular DNA origamis for intratracheal delivery to mice with ALI (Figure 3e).^[^ ^156^ ^]^ The origamis penetrated the lung mucus barrier, diffused to the lung epithelium (Figure 3f), and entered CD68^+^ alveolar macrophages (Figure 3g). Huang et al. intratracheally delivered DNA nanotubes that contain anti‐miR‐155 for reducing ALI.^[^ ^155^ ^]^ A portion of the Y strand of the nanotube has a sequence complementary to miRNA‐155, thereby acting as an antagonist of miR‐155 (Figure 3h). By flow cytometry, the authors showed that the nanostructure preferentially entered the alveolar macrophages and dendritic cells in the bronchoalveolar lavage fluid of ALI mice (Figure 3i), highlighting the self‐targeting ability of the nanostructure without targeting ligands.

Overall, there is now greater clarity over how different administration routes (i.v., i.p., intratracheal, and inhalation) govern the accumulation of nucleic acid nanostructures in the lungs. Some advanced studies featured quantitative measurements of cellular‐level distribution to various lung cell types, but most studies merely reported the organ‐level distribution data.

### 3.3 Liver

**Figure 4:**
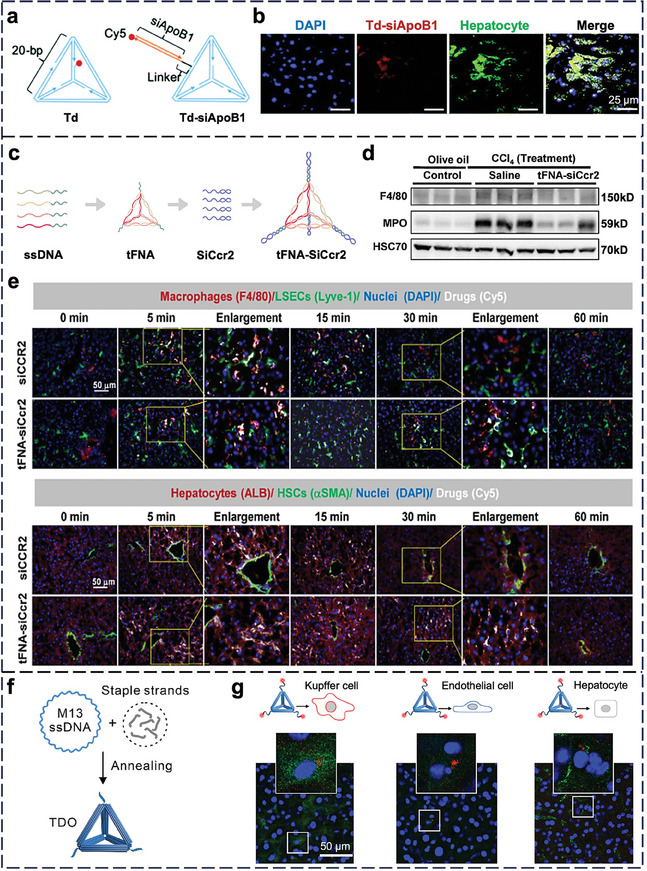
In vivo cell‐NP interaction of nucleic acid nanostructures in the liver. a) Preparation of siApoB1‐loaded DNA tetrahedron. ApoB1: apolipoprotein B1. b) Representative confocal images confirmed the colocalization of Cy5‐labeled siApoB1‐loaded DNA tetrahedron (red) with hepatocytes (green) 2 h post i.p. injection. Blue = nuclei (stained by DAPI). Reproduced with permission.^[^ ^157^ ^]^ Copyright 2024, Royal Society of Chemistry. c) Illustration of the assembly of tFNA‐siCcr2. d) Mice with liver cirrhosis were treated with tFNA‐siCcr2 or saline for 3 weeks. Immunocyte accumulation analysis was quantified by Western blotting. F4/80: marker of macrophages; myeloperoxidase (MPO): marker of neutrophils. Heat shock cognate protein 70 (HSC70) was used as a loading control. e) Uptake of tFNA‐siCcr2 by liver cells in vivo. The accumulation of Cy5‐labeled siCcr2 and Cy5‐labeled tFNA‐siCcr2 in macrophages (stained by F4/80), liver sinusoidal ECs (LSECs, stained by Lyve‐1, lymphatic vessel endothelial receptor 1), hepatocytes (stained by ALB, albumin) and hematopoietic stem cells (HSCs, stained by α‐SMA, α‐smooth muscle actin) in the murine liver was determined by immunofluorescence. Blue = nuclei (stained by DAPI). Reproduced with permission.^[^ ^159^ ^]^ Copyright 2023, American Chemical Society. f) Synthesis of Cy5‐labeled tetrahedral DNA origami. g) In vivo distribution of Cy5‐labeled tetrahedral DNA origami (red) to different liver cell types (green) 1 h post‐injection. Anti‐F4/80, anti‐CD31, and anti‐albumin were used to stain Kupffer cells, endothelial cells, and hepatocytes, respectively. Blue = nuclei (stained by DAPI). Reproduced with permission.^[^ ^160^ ^]^ Copyright 2024 Wiley‐VCH.

After traveling to the lungs, i.v. injected NPs return to the left ventricle of the heart via pulmonary veins and enter systemic circulation.^[^ ^137^ ^]^ Because i.v injected NPs often face sequestration by the liver, we investigated the cellular‐level distribution of SNAs in the healthy liver. After i.v. injecting miR‐146a‐SPIONs into plaque‐bearing mice, our flow cytometry data showed 4–6‐fold higher‐level association of miR‐146a‐SPIONs with total hepatic macrophages (Kupffer cells) than PEG‐SPIONs. Also, there was up to 4‐fold higher association of miR‐146a‐SPIONs to SR‐A‐rich hepatic macrophages than total hepatic macrophages (both SR‐A‐rich and SR‐A‐deficient inclusive). Consistent with our data in the aorta, we proved that the 3D miRNA shell of SNA promotes selective entry to SR‐A‐rich macrophages in the liver. Kim et al. attached siRNA against apolipoprotein B1, a marker for hypercholesterolemia, to a constituent strand of tile‐based DNA tetrahedron for intraperitoneal (i.p.) injection into healthy mice (**Figure** **4**a).^[^ ^157^ ^]^ The i.p. injection route permits NPs to reach the liver before systemic circulation, promoting liver accumulation. The tetrahedra entered hepatocytes (Figure 4b) and reduced blood cholesterol levels, yet it was unclear by what cellular mechanism they entered hepatocytes. Likewise, it was unknown if the tetrahedra entered other liver cell types responsible for the sequestration of NPs (Kupffer cells and ECs) just like miR‐146a‐SPIONs, rendering it difficult to assess the efficiency of delivery to hepatocytes.

Other researchers studied the interactions of nucleic acid nanostructures in the diseased liver. Hepatitis, a viral infection‐induced inflammation, can lead to liver fibrosis, cirrhosis, and liver failure. Chen et al. applied self‐therapeutic tFNAs to hepatic cells;^[^ ^158^ ^]^ in vitro, tFNAs enhance hepatocyte proliferation by activating Notch and Wnt signaling pathways and inhibit glucosamine‐induced apoptosis in the development of type 2 diabetes mellitus. Tian et al. loaded siRNA against C‐C chemokine receptor 2 (siCcr2), a key contributor to liver fibrosis and recruitment of immune cells, to tFNA by hybridization (Figure 4c).^[^ ^159^ ^]^ In mice with carbon tetrachloride (CCl_4_)‐induced liver fibrosis, siCcr2‐loaded tFNAs reduced profibrotic macrophages and neutrophils in the liver (Figure 4d). I.p. injected siCcr2‐loaded tFNAs entered hepatic macrophages and ECs (Figure 4e), consistent with our data on the intrahepatic cellular‐level distribution of SNAs.^[^ ^141^ ^]^ Zhu et al. i.v. injected 40‐nm tetrahedral DNA origamis (Figure 4f) into tumor‐bearing mice for studying their distribution.^[^ ^160^ ^]^ Confocal images depicted their localization in Kupffer cells, epithelial cells, and hepatocytes 1 h post‐injection (Figure 4g). It will be instructive to quantify the amounts of origamis in different liver cell types.

The liver is still a common destination organ for nucleic acid nanostructures, a phenomenon possibly attributable to the abundance of Kupffer cells that internalize such nanostructures via scavenger receptors. Access to hepatocytes remains a major delivery hurdle.

### 3.4 Kidney

**Figure 5:**
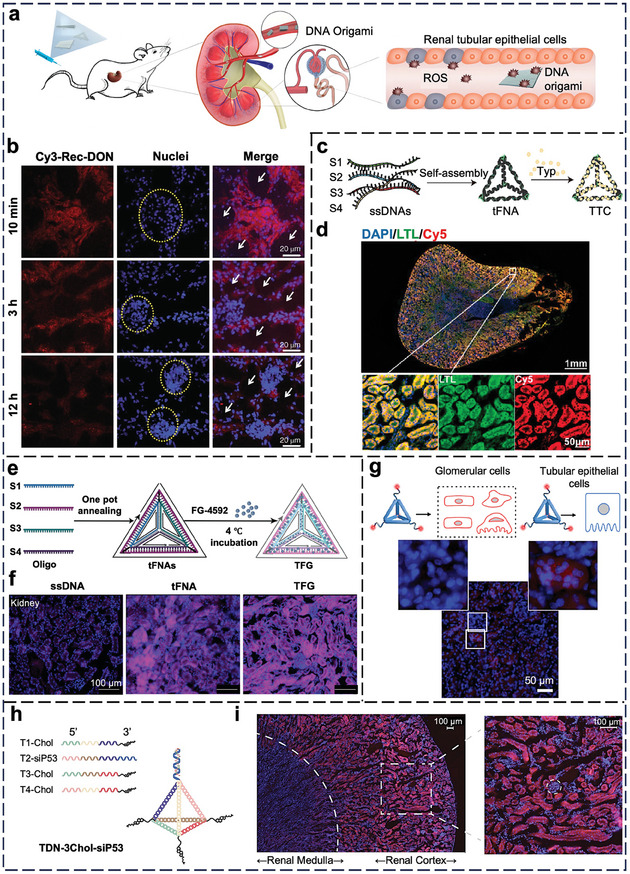
In vivo cell‐NP interaction of nucleic acid nanostructures in the kidneys. a) Selective kidney accumulation of DNA origami nanostructures (DONs) (rectangular, triangular, and tubular) and alleviation of AKI through ROS scavenging and reduction of oxidative stress. b) Confocal images of kidney sections confirmed the accumulation of Cy3‐labeled rectangular‐DON (red) in the kidney cells after i.v. injection. Blue = nuclei (stained by DAPI). Yellow dashed circles **=** glomeruli. White arrows **=** renal tubular lumina. Reproduced with permission.^[^ ^162^ ^]^ Copyright 2018, Springer Nature. c) Illustration of the assembly of tFNAs and tFNA‐Typ complex (TTCs). d) Representative confocal image indicates the targeting of renal tubules (labeled by LTL, lotus tetragonolobus lectin) by TTCs. Blue = nuclei (stained by DAPI). Reproduced with permission.^[^ ^164^ ^]^ Copyright 2023, American Chemical Society. e) Preparation of FG‐4592‐loaded tFNAs (TFG). f) Distribution of Cy5‐labeled ssDNA, tFNA, and TFG to kidney tubules. Blue = nuclei (stained by DAPI). Reproduced with permission.^[^ ^165^ ^]^ Copyright 2024, Wiley‐VCH. g) Representative confocal image confirmed the predominant distribution of Cy5‐labeled tetrahedral DNA origamis (red) to tubular epithelial cells 1 h post‐injection. Blue = nuclei (stained by DAPI). Reproduced with permission.^[^ ^160^ ^]^ Copyright 2024, Wiley‐VCH. h) Illustration of cholesterol‐modified tetrahedral DNA for delivering siP53 (TDN‐3Chol‐siP53). i) Representative confocal images depicted the preferential distribution of Cy3‐labeled TDN‐3Chol‐siP53 (red) to the tubular cells near the glomerulus (in circle). Blue = nuclei (stained by DAPI). Reproduced with permission.^[^ ^166^ ^]^ Copyright 2024, Wiley‐VCH.

The kidneys determine in vivo distribution of i.v. injected NPs. The glomerular filtration barrier, consisting of a fenestrated endothelium, glomerular basement membrane, and podocytes, regulates the passage of NPs from blood to urine with a cutoff size of ≈10 nm. Therefore, delivery to kidney tubule cells requires passage through the glomerular filtration barrier.^[^ ^29^ ^]^ Thai et al. prepared small DNA tetrahedra (≈6 nm) using four different types of sugar backbone modification: D‐DNA, L‐DNA (the mirror image of the naturally occurring D‐DNA), 2′‐methoxy‐RNA, and 2′‐fluorine‐RNA.^[^ ^161^ ^]^ All four tetrahedra have an edge length of only 10 base pairs to keep the overall size <10 nm for glomerular filtration but with sufficient DNA duplexes (melting point >37 °C) for maintaining stable assembly under physiological conditions. Upon i.v. injection into healthy mice, only L‐DNA‐based tetrahedra accumulated inside the kidney tubule cells without pronounced sequestration by the liver; D‐DNA‐based tetrahedra showed limited accumulation in the kidney possibly due to in vivo degradation, whereas the tetrahedra modified with 2′‐methoxy‐RNA and 2′‐fluorine‐RNA localized in the liver due to adsorption by serum proteins and sequestration by hepatic macrophages. By contrast, larger nucleic acid nanostructures necessitate glomerular disassembly to access tubules. Jiang et al. i.v. injected large DNA origami nanostructures of three different shapes (rectangle 90 × 60 nm; triangle with an edge length of 120 nm, and tube with a length of 400 nm) into healthy mice (**Figure** **5**a),^[^ ^162^ ^]^ but they detected insignificant effect of shape on kidney accumulation possibly because in vivo disassembly into individual oligonucleotides masked the effect of the initial origami shape. The nanostructures localized in the glomeruli before entering tubule cells (Figure 5b), consistent with polymer‐siRNA NPs.^[^ ^163^ ^]^

Recent investigations shed light on how nucleic acid nanostructures interact with cells in diseased kidneys. For instance, acute kidney injury (AKI) is marked by deteriorated renal function causing the accumulation of metabolic wastes (e.g., blood urea nitrogen and creatinine). Yan et al. employed a typhaneoside (Typ)‐encapsulated DNA tetrahedron for AKI treatment (Figure 5c).^[^ ^164^ ^]^ The DNA nanostructures selectively enter tubule cells of AKI over healthy kidneys (Figure 5d), possibly because the retarded fluid flow in the injured kidney provides more opportunities for NPs to interact with tubule cells. Similarly, Chen et al. designed DNA tetrahedra for intercalating FG‐4592, a drug inhibitor of hypoxia‐inducible factor prolyl‐hydroxylase with kidney protective properties (Figure 5e), and i.v. injected the nanostructure into mice with AKI.^[^ ^165^ ^]^ This ≈20‐nm tetrahedra‐drug complex was localized to kidney tubules in healthy mice 24 h post‐injection (Figure 5f) and alleviated the renal tubular injury in AKI mice. It would be instructive to address how AKI affects the kidney delivery of this nanostructure. Classical 40‐nm tetrahedral DNA origamis (as described in the previous section) accumulated in tubular epithelial cells rather than passing through the glomerular filtration barrier (Figure 5g).^[^ ^160^ ^]^ Li et al. designed a tFNA with cholesterol chains at its three vertices for i.v. delivery of siRNA against p53 (which mediates kidney injury) in mice (Figure 5h). Cholesterol modification prolonged blood circulation and promoted delivery to tubules in healthy mice (Figure 5i). The siRNA‐loaded tFNA prevented tubular apoptosis in AKI mice.^[^ ^166^ ^]^ These results were surprising because they pronouncedly depart from the established understanding in the nanomedicine field that the renal filtration size cutoff is typically 10 nm, but unfortunately, the authors did not provide a plausible explanation.

In short, researchers have cherished the importance of compact nucleic acid nanostructures for passing the glomerular filtration barrier and accessing kidney tubules, showing a deeper understanding of tissue‐level distribution. Yet, few studies proved their delivery to specific kidney cell types, making it difficult to determine whether they would accumulate in the kidney (critical for therapeutic use) or face rapid clearance to the bladder. Adding ligands (small molecules or antibodies) to target different kidney cell types (e.g., tubules and mesangial cells) will enhance the overall efficiency of kidney delivery.

### 3.5 Brain

**Figure 6:**
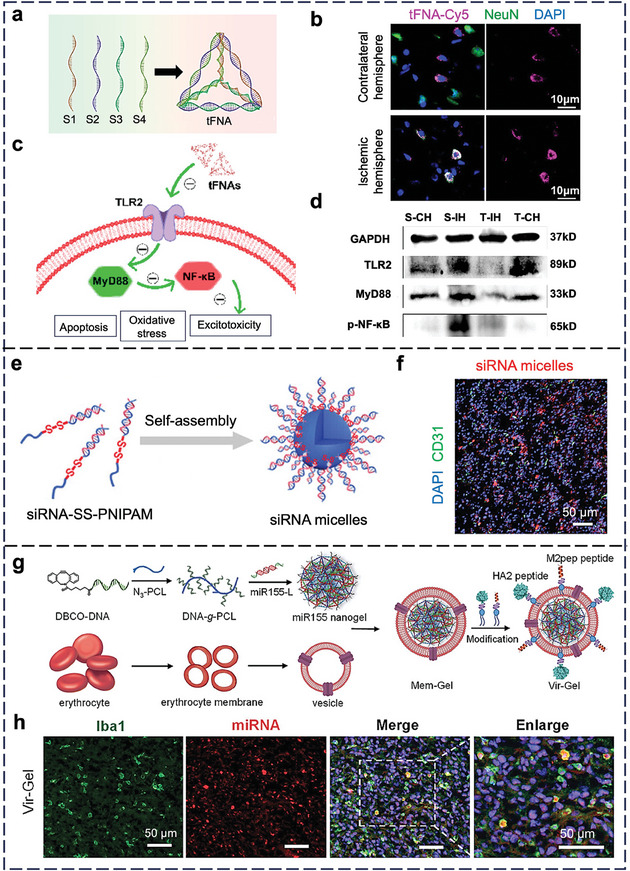
In vivo cell‐NP interaction of nucleic acid nanostructures in the brain. a) Synthesis of tFNAs. b) Uptake of tFNAs by neurons in the brain tissue of rats with ischemic stroke. Pink = tFNA‐Cy5; gree*n =* neurons (labeled by NeuN); blue = nuclei (stained by DAPI). c) The protective effects of tFNAs on the ischemic brain stem from the downregulation of the TLR2 signaling pathway and subsequent interference with the ischemia cascades in ischemic stroke. d) Protein expression of TLR2, MyD88, and p‐NF‐κB in the brain tissue of tMCAO rats. S‐CH and S‐IH denote contralateral hemisphere, and ischemic hemisphere in saline‐treated rats with ischemic stroke, respectively. T‐CH and T‐IH denote the contralateral hemisphere and ischemic hemisphere in tFNA‐treated rats with ischemic stroke, respectively. Glyceraldehyde 3‐phosphate dehydrogenase (GAPDH) was used as a loading control. Reproduced with permission.^[^ ^167^ ^]^ Copyright 2022, American Chemical Society. e) Preparation of siRNA micelle. PNIPAM: poly(N‐isopropylacrylamide). f) Confocal images show the accumulation of Cy5‐labeled siRNA micelles (red) in brain tumor slices 10 h post‐injection. Blue = nuclei (stained by DAPI); gree*n =* blood vessels (stained by CD31). Reproduced with permission.^[^ ^168^ ^]^ Copyright 2021, Wiley‐VCH.​ g) Construction of peptide‐modified and erythrocyte membrane‐coated Vir‐Gel. DNA‐g‐PCL: DNA‐grafted polycaprolactone. h) Confocal images of brain slices after treatment with Vir‐Gel show the accumulation of miR155‐L (labeled by Cy5.5) near microglia and macrophages (Iba1). Scale bars: 50 µm. Reproduced with permission.^[^ ^169^ ^]^ Copyright 2021, Wiley‐VCH.

The blood‐brain barrier (BBB), a monolayer of brain ECs, poses a challenge to deliver i.v. injected NPs into the brain. Zhou et al. showed that self‐therapeutic tFNAs protect against neuronal apoptosis in rats with ischemic stroke (IS) (**Figure** **6**a), marked by the blockage of the artery leading to the brain.^[^ ^167^ ^]^ After i.v. injection, tFNAs entered neurons in the contralateral and ischemic hemispheres (Figure 6b) and protected the neurons from apoptosis by inhibiting the TLR2, MyD88, and p‐NF‐ĸB along the TLR2 pathway in the brain (Figure 6c,d). Jiang et al. reported ≈40‐nm micelle‐cored SNAs via self‐assembly of a diblock copolymer of siRNA and poly(N‐isopropylacrylamide) for i.v. delivering siRNA against signal transducers and activators of transcription 3 (STAT3, a marker for cell proliferation) to the brain (Figure 6e).^[^ ^168^ ^]^ The authors detected cellular entry of SNAs in brain slices (Figure 6f) and reduced tumor growth in vivo, but the cellular‐level distribution of SNAs in the brain parenchyma and tumor tissue was unclear, nor was the selectivity toward the tumor part of the brain. Gao et al. developed DNA/RNA hybrid nanogels by using miR‐155 (which inhibits inflammatory proteins in the brain microglia and macrophages) to crosslink a DNA‐grafted polycaprolactone polymer brush (Figure 6g).^[^ ^169^ ^]^ The authors coated the nanogels with an erythrocyte membrane to prolong blood circulation and conjugated M2pep and HA2 peptides to improve cellular targeting and promote fusion with the endosomal membrane for gene release. Despite its large size of 154 nm, the nanogel entered microglial cells and macrophages upon i.v. injection into mice with glioblastoma (Figure 6h). The question is whether the pores on the compromised BBB due to glioblastoma are large enough for the nanogel to penetrate; if not, was the disassembly of the nanogel en route required?

In summary, researchers have now postulated that penetration of BBB depends on the disease stage and may involve receptor‐mediated transcytosis,^[^ ^170^ ^]^ but direct in vivo evidence of entry to various brain cell and receptor types is often missing. Rather, most studies featured merely the localization of nucleic acid nanostructures in the brain tissue.

### 3.6 Tumor

Tumors are the most frequently studied disease tissues, accounting for ≈48% of publications in the in vivo application of nucleic acid nanostructures over the past 5–6 years. However, past publications focused on NP design and efficacy evaluation, with distribution studies merely confined to the organ level. Below, we will review the latest interactions of nucleic acid nanostructures with tumor‐associated cell types.

**Figure 7:**
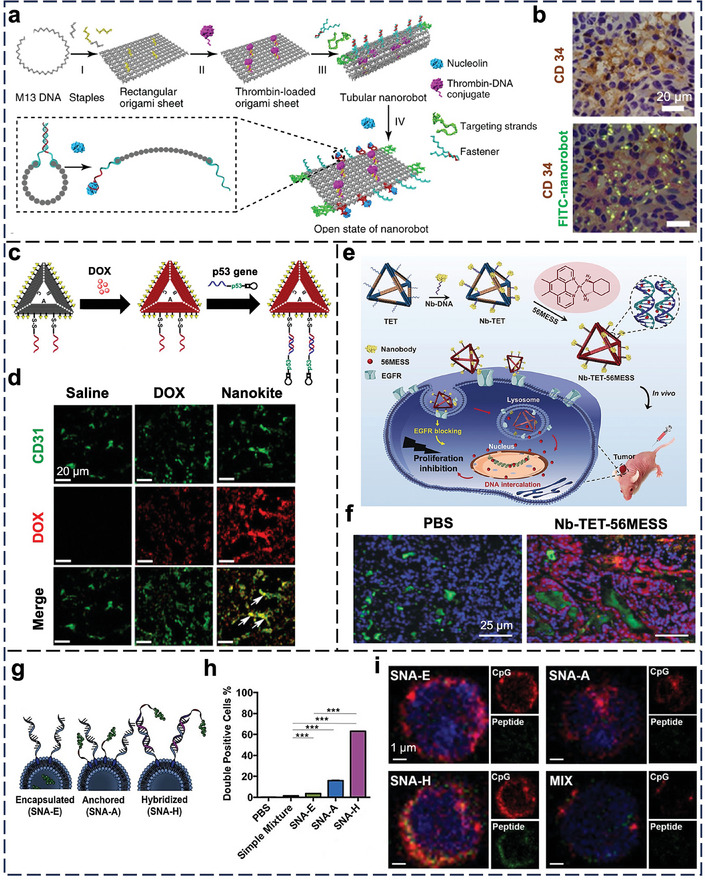
In vivo cell‐NP interactions of nucleic acid nanostructures in the tumor. a) Illustration of the thrombin‐loaded DNA nanorobot construction, and its reconfiguration in response to nucleolin binding. b) Confocal images show the colocalization of FITC‐labeled nanorobots (green) with blood vessel‐rich regions (anti‐CD34; brown). Blue = nuclei. Reproduced with permission.^[^ ^171^ ^]^ Copyright 2018, Springer Nature.  c) Structure design of DNA origami nanokite. d) Confocal images showed the colocalization of tumor blood vessels (anti‐CD31; green) and DOX (red) at 24 h post i.v. injection. White arrows indicate DOX around blood vessels. Reproduced with permission.^[^ ^83^ ^]^ Copyright 2018, American Chemical Society. e) Illustration of a nanobody‐conjugated DNA tetrahedron. f) Accumulation of Cy5‐labeled DNA tetrahedra (red) in the tumor. Blood vessels were labeled by anti‐CD31 (green). Blue = nuclei (stained by DAPI). Reproduced with permission.^[^ ^172^ ^]^ Copyright 2019, Wiley‐VCH. g) Illustration of SNA‐E, SNA‐A, and SNA‐H. h) Fraction of cells showing high levels of both CpG and OVA1, recovered from the DLNs of mice 2 h following s.c. injection, as determined by flow cytometry. A simple mixture denotes the physical mixture of adjuvant and antigen. i) Confocal images of cells recovered from DLNs from mice 4 h following immunization. Gree*n =* OVA1 peptide labeled with tetramethyl‐rhodamine; red = Cy5‐CpG. Reproduced with permission.^[^ ^173^ ^]^ Copyright 2019, National Academy of Sciences.

ECs constitute the blood vessels around tumors to supply oxygen and nutrients. Li et al. reported a DNA origami nanotube (90 nm × 19 nm) that contains thrombin as the drug to trigger thrombosis (which deprives the tumor of nutrients) in its core, DNA aptamers to target nucleolin on tumor vessels on its surface, and fastener strands that relax the nanotube to its original rectangular origami structure upon contact with nucleolin (**Figure** **7**a).^[^ ^171^ ^]^ Upon i.v. injection into orthotopic tumor‐bearing mice, the nanotubes entered CD34‐expressing ECs (Figure 7b) and released thrombin to induce vessel infarction and tumor necrosis. Liu et al. i.v. injected a triangle DNA origami nanokite to deliver linear tumor suppressor gene p53 and DOX to mice bearing multidrug‐resistant tumors (Figure 7c).^[^ ^83^ ^]^ The nanokites associated with ECs in the tumor (Figure 7d), but there was no proof whether they reached the tumor cells where the cargoes should exert their anti‐cancer efficacy.

Tumor cell is a major tumor cell type, so perturbation of tumor cells is a common anti‐tumor strategy. Wu et al. conjugated nanobodies against epidermal growth factor receptor (EGFR; a marker of tumor cells) and intercalated water‐soluble platinum‐based drug molecules to a double‐bundle DNA tetrahedron (Figure 7e).^[^ ^172^ ^]^ Similarly, the authors proved the i.v. delivery of the tetrahedra to the A431 tumor xenograft model (Figure 7f) and detected tumor inhibition. It was unclear if they preferentially entered EGFR‐positive tumor cells over other cell types and then escaped from the lysosome for cytosolic delivery.

Priming the immune system to attack tumor cells is another therapeutic approach. Wang et al. reported three types of SNAs that are compositionally identical yet structurally distinct from cancer vaccines.^[^ ^173^ ^]^ They all share a liposome core, a shell of CpG oligonucleotides as agonists of TLR9, and OVA‐1 peptides as antigens. Yet, the locations of the antigen are different, either encapsulated inside the liposome core (E), conjugated to the liposome surface via cholesterol groups (A), or hybridized to the shell of CpG oligonucleotides (H) (Figure 7g). Upon s.c. injection into mice, flow cytometry analysis of the draining lymph nodes (DLNs) revealed that SNA H had the highest uptake by CD11c^+^ dendritic cells (Figure 7h). Confocal images showed similar levels of CpG oligonucleotides delivered by each SNA structure, but the amount of OVA‐1 delivered by SNA H was the largest (Figure 7i). Accordingly, vaccination by SNA H induced the most effective antigen‐specific cytotoxic T lymphocyte responses and eradication of cancer cells. Qi et al. programmed the self‐assembly of a long ssRNA molecule into a rectangular RNA origami nanostructure that surprisingly stimulated an innate immune response via TLR3 without CpG oligonucleotides, lipids, or other agonists in vitro,^[^ ^174^ ^]^ a finding supported by the activation of CD40 (a co‐stimulatory molecule on activated immune cells) and CD86 (a marker of antigen‐presenting cells). The RNA origami retarded tumor growth after i.p. injection into mice with metastatic colon cancer, but in vivo evidence of TLR3 activation was unavailable.

Most reports predominantly alluded to the “enhanced permeability and retention (EPR)” effect as the mechanism for tumor entry. EPR was originally reported based on proteins that were at most 160 kDa in molecular weight,^[^ ^175^ ^]^ so many nucleic acid nanostructures are pronouncedly larger and exhibit much slower diffusion from the blood vessel and in the tumor tissue. Recent studies on solid inorganic NPs even pointed to an active mode of extravasation through the tumor endothelial cells.^[^ ^176^ ^]^ For nanostructures that contain tumor‐targeting ligands, direct evidence of in vivo uptake by a specific tumor cell type is not available. As a result, it is difficult to objectively assess the efficiency of tumor active targeting.

### 3.7 Skin

The stratum corneum, the outermost surface layer of dead cells, is a critical lipid‐containing layer that obstructs topical delivery to cells in the viable skin underneath. Examples of lipids in the stratum corneum are ceramides, cholesterol, and free fatty acids. In general, a smaller NP size (<20 nm) and higher lipid content favor penetration of stratum corneum.^[^ ^28^ ^]^ While nucleic acid nanostructures can be made small enough for penetration, their hydrophilic DNA or RNA building blocks are incompatible with the hydrophobic stratum corneum; therefore, cream or other types of excipients are needed. Still, nucleic acid nanostructures are attractive gene carriers because, in the presence of cream, they can enter skin cells without using transfection agents, but single‐stranded oligonucleotides typically do not.

**Figure 8:**
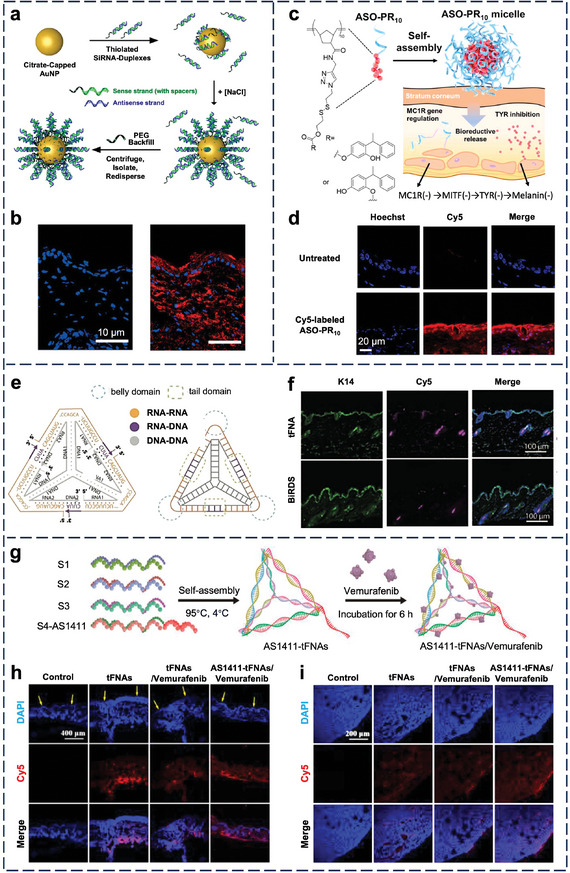
In vivo cell‐NP interaction of nucleic acid nanostructures in the skin. a) Synthesis of siRNA‐based SNAs. b) Penetration and clearance of SNAs in hairless mouse skin 3 h after application. Blue = nuclei (stained by DAPI). Red = Cy5‐labeled siRNA‐loaded SNAs. Reproduced with permission.^[^ ^94^ ^]^ Copyright 2012, National Academy of Sciences. c) Schematic illustration of skin depigmentation of micellar SNAs (ASO‐PR_10_ micelle) composed of antisense oligonucleotides (ASOs) and phenylethyl esorcinol (PR). ASO targets melanocortin 1 receptor (MC1R); PR is a potent tyrosinase (TYR) inhibitor. MITF: microphthalmia‐associated transcription factor, which transcriptionally regulated TYR. d) Confocal images of mouse ear sections after treatment with Cy5‐labeled ASO‐PR_10_ (red). Reproduced with permission.^[^ ^115^ ^]^ Copyright 2021, American Chemical Society. e) Structure design of the tFNA‐based bioswitchable miR inhibitor delivery system (BiRDS) and the position of the belly domain and the tail domain. f) Confocal images of skin tissue after 1 d of topical administration of the miR inhibitor, tFNA, and BiRDS. K14: keratin 14, a biochemical marker expressed in the epidermis and hair follicle. Scale bar: 100 µm. Reproduced with permission.^[^ ^179^ ^]^ Copyright 2022, Wiley‐VCH.​ g) Hybridizing AS1411 and loading vemurafenib to tFNA. AS1411, a DNA aptamer, binds to nucleolin on the tumor cell membrane. h, i) Distribution of Cy5‐labeled AS1411‐tFNAs/vemurafenib (red) in the h) skin and i) s.c. tumor upon topical application. Blue = nuclei (stained by DAPI). Arrows indicate the stratum corneum. Reproduced with permission.^[^ ^180^ ^]^ Copyright 2024, Elsevier.

In healthy mice, siRNA‐based, gold‐cored SNAs (**Figure** **8**a) penetrated the stratum corneum and entered cells in the epidermis and dermis (Figure 8b).^[^ ^94^ ^]^ In mice with diabetes‐induced wound injury, similar 30‐nm SNAs inhibited ganglioside‐monosialic acid 3 synthases overexpressed in diabetic mice and reversed diabetes‐induced wound injury. The wounded skin should reduce the barrier for skin permeation, but the authors did not evaluate the effect of wound formation on entry to epidermal cells relative to healthy skin.^[^ ^177^ ^]^ Polymer‐cored SNAs (Figure 8c) also entered epidermal cells and reached the dermis (Figure 8d). The 20‐nm micellar SNAs stem from the assembly of a bifunctional amphiphile made by click coupling of an antisense oligonucleotide to a polymer with many small molecule prodrugs.^[^ ^115^ ^]^ Surprisingly, larger 100‐nm siRNA‐based, hyaluronic acid‐cored SNAs entered epidermal cells upon topical application on skin with hypertrophic scar composed of excessive deposits of collagen, but the mechanism for skin penetration is unclear.^[^ ^178^ ^]^

Like SNAs, tFNAs also permeate the stratum corneum to reach the dermis, but the routes of skin permeation between the two types of nanostructures differ. While SNAs adopt a transcellular route across the skin (because they enter epidermal cells), tFNAs adopt transappendageal penetration. Li et al. designed tFNAs by wrapping three identical miR inhibitor strands around a nucleic acid core and extending the 3′ end of the miR with four nucleotides to form an RNase H‐responsive DNA‐RNA hybridized tail domain (Figure 8e).^[^ ^179^ ^]^ Upon contact with intracellular RNaseH, the tFNA changes its configuration from 3D to 2D to foster the release of miRNA. After topical application on the skin of mice, the tFNAs (≈11 nm in size) accumulated primarily in hair follicles and sweat glands (Figure 8f), although it was unclear in which skin cell type the tFNAs resided or why the transcellular route was not adopted by tFNAs with their small size. Similarly, Xiao et al. connected DNA aptamers AS1411 (with a high affinity for nucleolin expressed on the tumor cells) at the apex of tFNAs and loaded vemurafenib, a common small molecule inhibitor of the B‐RAF protein (Figure 8g).^[^ ^180^ ^]^ Upon topical delivery into mice with s.c. melanoma, this tFNA crossed the basal layer of skin (Figure 8h) and diffused to the tumor core at a depth of 300–400 µm from the surface (Figure 8i). This targeted, drug‐loaded tFNA downregulated mutant BRAF kinase and induced melanoma cell apoptosis. Recently, tFNAs were used to deliver miR‐125b, one of the most downregulated miRNAs in psoriasis skin, to treat imiquimod‐induced psoriasis in mice.^[^ ^181^ ^]^ Free miRNA‐125 was collected in hair follicles and stratum corneum and, to a lesser extent, in the epidermis, but miR‐125b‐loaded tFNA was more abundantly and homogenously distributed to the epidermis.

Overall, tFNAs are promising drug delivery systems for skin penetration and treating skin diseases, but little is known about their entry into various skin cell types. Interestingly, self‐therapeutic tFNAs showed efficacy when administered beneath the skin. For instance, upon s.c. injection into rats with cutaneous wounds, tFNAs reduced tissue fibrosis and proinflammatory cytokines by activating the Akt‐signaling pathway inside skin cells.^[^ ^182^ ^]^

### 3.8 Bone

Bones offer protection and mechanical support, facilitate movement, contribute to blood production and homeostasis, and house progenitor cells (mesenchymal and hemopoietic).^[^ ^183^ ^]^ Bone diseases and skeletal disorders may hinder mobility and cause pain and even mortality, such as arthritis, osteoarthritis, and bone cancer, few effective treatments are available.

**Figure 9:**
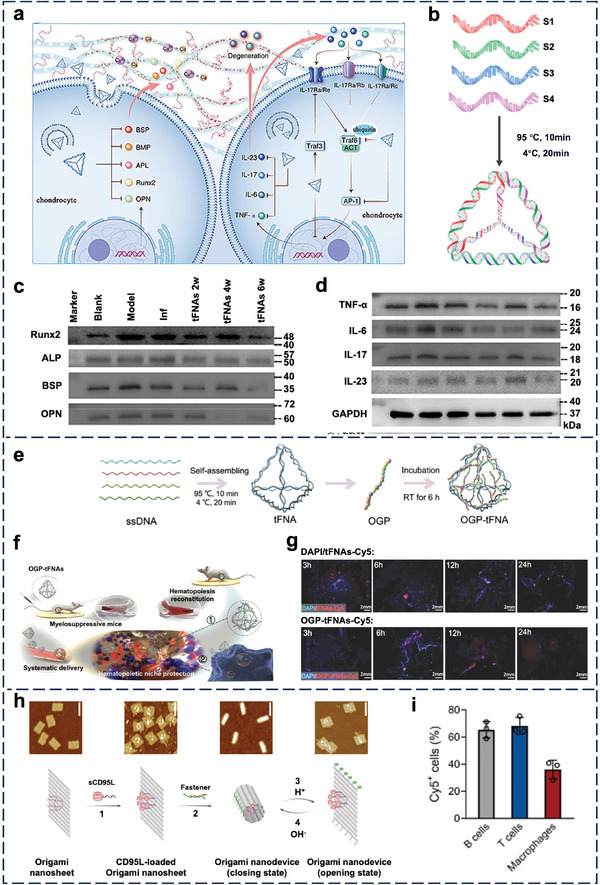
In vivo cell‐NP interactions of nucleic acid nanostructures in the bone. a) Illustration of self‐therapeutic tFNAs that regulate the IL‐17 pathway to alleviate inflammation and inhibit heterotopic ossification in ankylosing spondylitis. b) Synthesis of tFNAs. c‐d) Western blot detection of osteogenesis‐related proteins and inflammatory proteins in the intervertebral disc tissue. Reproduced with permission.^[^ ^184^ ^]^ Copyright 2023, American Chemical Society. e) Preparation of OGP‐tFNAs. OGP: osteogenic growth peptide. f) Systematic delivery of OGP‐tFNAs and its effect on hematopoiesis reconstitution. OGP: osteogenic growth peptides. g) In vivo distribution of tFNAs and OGP‐tFNAs after tail vein injection. Blue = DAPI; Red = Cy5‐labeled tFNAs. Reproduced with permission.^[^ ^185^ ^]^ Copyright 2022, Wiley‐VCH. h) Construction of the DNA origami nanosheet with a hexagonal CD95L array pattern and pH‐responsive structural transformation to a nanotube. i) Quantification of Cy5‐positive synovial cell subsets of collagen‐induced arthritis mice 4 h post i.v. injection of Cy5‐labeled DNA origamis by flow cytometry. Reproduced with permission.^[^ ^187^ ^]^ Copyright 2024, Springer Nature.

Wang et al. i.p. injected self‐therapeutic tFNAs (**Figure** **9**a,b) into mice with ankylosing spondylitis induced by the human versican core protein. While the authors detected the in vivo inhibition of osteogenic‐related and IL‐17 signaling proteins (Figure 9c,d), they did not study the cellular‐level distribution of NPs in the spinal cord.^[^ ^184^ ^]^ Zhang et al. i.v. injected tFNAs that contained osteogenic growth peptides (Figure 9e) into mice with cyclophosphamide‐induced myelosuppression (Figure 9f).^[^ ^185^ ^]^ Confocal images of the bone marrow smear showed the localization of peptide‐loaded tFNAs in the bone marrow (Figure 9g). Although it was unclear which cell types tFNAs entered, there was upregulation of hematopoietic stem cells (HSC) markers essential to self‐renewal and development. Li et al. utilized tFNAs to i.p. deliver curcumin, an agent that regulates ferroptosis‐mediated cell death, to mice with diabetic osteoporosis. The curcumin‐loaded tFNA upregulated the nuclear factor erythroid 2‐related factor 2 (NRF2)/glutathione peroxidase 4 (GPX4) pathway in bone marrow cells, enhancing osteogenesis.^[^ ^186^ ^]^ Recently, Li et al. patterned CD95 ligands hexagonally to a rectangular DNA origami (Figure 9h).^[^ ^187^ ^]^ The ligands were <10 nm apart to mirror the spatial arrangement of CD95 receptors on the immune cell surface. Initially closed at a neutral pH, the ligand‐patterned origami became open at an acidic pH (6.5) characteristic of the inflamed synovial tissue (Figure 9h). Upon i.v. injection to mice with collagen‐induced arthritis, the ligand‐patterned origami associated with different synovial cells, including >60% of the T and B cells (Figure 9i). Overall, i.v. or i.p. delivery to bones is challenging because of their poor blood perfusion,^[^ ^188^ ^]^ requiring high doses that may induce toxicity. Local delivery to the bone joint is garnering attention as a low‐dose alternative. Shi et al. injected self‐therapeutic DNA tetrahedra into the knee joint cavity of rats with osteoarthritic knees.^[^ ^189^, ^190^ ^]^ The DNA tetrahedra inhibited apoptosis and upregulated chondrogenic markers (collagen type 2 and aggrecan) in the articular cartilage cells, leading to cartilage repair. In short, nucleic acid nanostructures are promising drug carriers to the bone or self‐therapeutic agents for bone diseases, but our knowledge of their cellular‐level distribution studies in the bone is still limited.

### 3.9 Non‐mammalian Species

Studies on non‐mammalian species will yield basic knowledge or biological imaging rather than disease treatment, so it is understandable that their focus does not necessarily center on or prove whether the findings are translatable to higher‐order mammals.

**Figure 10:**
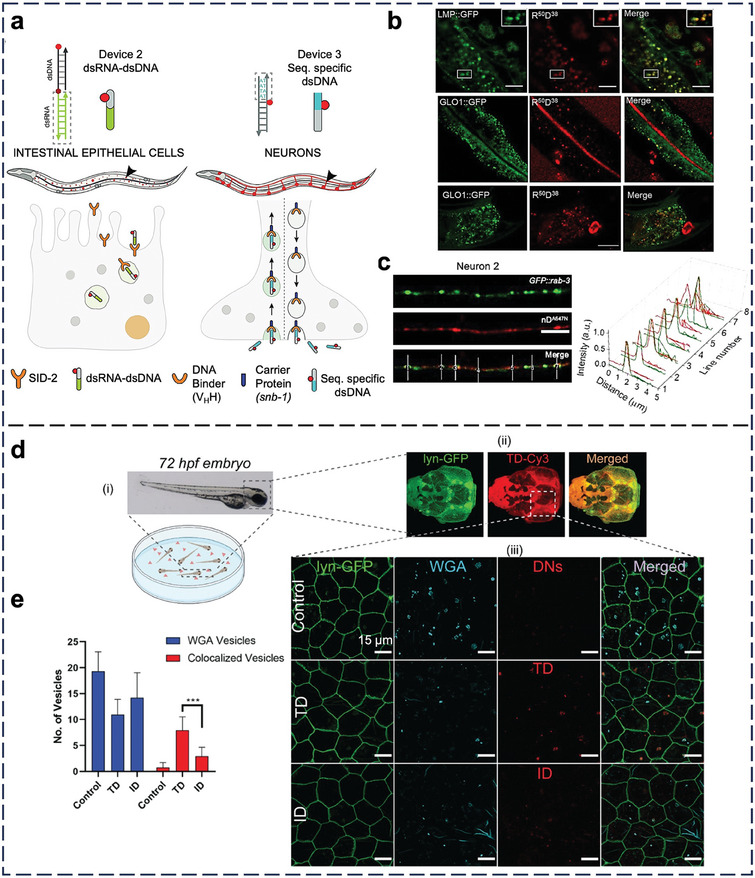
In vivo cell‐NP interactions of nucleic acid nanostructures in non‐mammalian species. a) Schematic illustration of strategies for targeting DNA devices to ECs and neurons in C. elegans. b) Representative images of colocalization between lysosome‐related organelle markers (LMP::GFP and GLO‐1::GFP, green) and R^50^D^38^ nanostructures (red) in intestinal epithelial cells. c) Representative images of 9E‐modified DNA nanostructures (nD^A647N^, red) uptake by neurons (left). GFP::rab‐3 denotes endosomes (green). Normalized line intensity profiles of regions of interest indicated in the merged image are plotted (right). Reproduced with permission.^[^ ^191^ ^]^ Copyright 2021, Chakraborty et al.​ d) In vivo uptake of DNA nanostructures by zebrafish embryo. i) Schematic illustration of experimental design. 72 hpf (hours post fertilization) zebrafish embryos were soaked in a medium containing DNA nanostructures and incubated for 4 h. ii) Confocal images of the dissected head of 72 hpf embryo show the internalization of Cy3‐labeled DNA tetrahedra (red). The zebrafish embryos were obtained from a transgenic line in which the expression of lynEGFP is driven by *claudin* promoter marking the membrane of peridermal cells. Gree*n =* membrane‐tethered lynEGFP. iii) Confocal images of the peridermal cells of show the internalization of DNA tetrahedra. DNs: DNA nanostructures; TD: tetrahedron; ID: icosahedron. e) Quantification of DNs internalized by peridermal cells, based on the number of DN vesicles colocalized with wheat germ agglutinin (WGA) vesicles, a marker of endocytosis. Reproduced with permission.^[^ ^192^ ^]^ Copyright 2022, American Chemical Society.

Due to their transparent appearance and ease of genetic manipulation, zebrafish and worms are now utilized for investigating the cell‐NP interactions of nucleic acid nanostructures. Chakraborty et al. designed a DNA‐RNA hybrid nanostructure (R^50^D^38^) with a 38‐bp double‐stranded DNA (dsDNA) linked to a 50‐bp double‐stranded RNA (dsRNA) (**Figure** **10**a). After loading the nanostructures to the intestinal lumen of Caenorhabditis elegan worms, the authors detected their association with the systemic RNA interference defective protein 2 (SID‐2), a Caenorhabditis‐specific scavenger receptor, on intestinal ECs and their localization to the lysosome inside these cells (Figure 10b). Separately, upon injection of the nanostructure modified with a synthetic receptor (9E) into the pseudocoelom, authors detected its localization in endosomes of neurons (Figure 10c) that express synaptobrevin‐1 (which mediates vesicle priming), possibly because neurons do not express either scavenger receptors or SID proteins.^[^ ^191^ ^]^ Rajwar et al. studied the effect of NP shape on cellular entry using zebrafish embryos that were genetically engineered to express the enhanced green fluorescent protein (EGFP) on the membrane of peridermal cells. After soaking the zebrafish with DNA nanostructures, the authors observed that tetrahedra more effectively penetrated the mucus barrier and entered peridermal cells in the squamous epithelium (the most superficial embryonic layer) than icosahedra (Figure 10d,e), although the mechanism for the shape‐dependent uptake in vivo is unclear.^[^ ^192^ ^]^

## 4 Studies on Large Animals and Humans

It is desirable to portray the emerging trend of using nucleic acid nanostructures in large animals as the field looks forward to clinical translation,^[^ ^193^ ^]^ but such studies remain infrequent. In Section 4, we slightly relax our search criteria from those in Section 3 by also featuring the safety and efficacy of nucleic acid nanostructures; we only mention in vivo cell‐nano interactions when such data are available.

### 4.1 Rabbit

Rabbits are common medium‐sized animals between rodents and non‐human primates. Their advantages are availability, relatively low cost given their short life cycles, ease of handling, and compatibility with genetic manipulation.

Rabbit eyes are suitable for eye research because they are similarly sized to human eyes.^[^ ^194^ ^]^ Liu et al. reported the treatment of corneal injuries using self‐therapeutic tFNAs upon application via eye drops on rabbit models with alkali‐burned injury.^[^ ^195^ ^]^ The tFNAs reduced corneal opacity and accelerated epithelial healing, thereby leading to eye repair. The authors did not prove their delivery to the eye cells in vivo, nor did they explain the in vivo mechanism for the self‐therapeutic tFNAs in the eyes. Their in vitro data suggested that tFNAs enhanced the proliferation and migration of human corneal epithelial cells, possibly by upregulating the phosphorylation of extracellular signal‐regulated kinase 1/2 (ERK1/2) and p38.

Rabbits are appealing models for bone research with similar bone metabolism and remodeling capability between rabbits and humans.^[^ ^196^ ^]^ Li et al. utilized DNA tetrahedra to deliver miR‐335‐5p, a regulator of osteogenic differentiation of bone mesenchymal stem cells.^[^ ^197^ ^]^ When incorporated into a hydrogel for intramuscular injection into rabbits with steroid‐associated osteonecrosis, the hybrid DNA‐RNA tetrahedra repaired bone defects and caused the formation of new bones. The authors did not identify the bone cell types that internalized the tetrahedra, but they detected an enhanced level of β‐catenin in the bones, suggesting an upregulation of the Wnt pathway for bone remodeling and regeneration. The DNA tetrahedra without miR‐335‐5p also promoted osteogenesis on rabbit‐derived bone mesenchymal stem cells in vitro, but the authors did not validate their effect in vivo.

Adult rabbits are utilized for studying the efficacy of cartilage repair because their cartilages lose their self‐healing capacity.^[^ ^198^ ^]^ Upon injection of self‐therapeutic tFNAs into the knee joint cavity of rabbits with cylindrical cartilage defects, Fu et al. detected more CD73^+^ and CD105^+^ cells in the regenerated tissue.^[^ ^199^ ^]^ The result implies the migration of synovial mesenchymal stem cells to the injured site to cause tissue repair. tFNAs promoted the chondrogenic differentiation of synovial stem cells by upregulating the phosphorylation of small mothers against decapentaplegic 2/3 (Smad2/3) in vitro, but in vivo evidence was missing.

### 4.2 Pig

Pigs have similar hemodynamics, cardiovascular anatomy, and lipid metabolism to humans,^[^ ^193^ ^]^ so studies on pigs offer insights into the safety of NPs in the cardiovascular system. Recall in Section 3.6 the DNA origami nanotube for treating cancer by inducing thrombosis of tumor blood vessels in mice.^[^ ^171^ ^]^ To validate its safety in non‐rodents, the authors showed that the nanotube did not change blood coagulation parameters or morphology of cardiovascular tissues in healthy Bama minipigs (8–10 months old) after injection via the marginal ear vein.

### 4.3 Non‐human Primate

Non‐human primates are established species for testing the toxicity of nucleic acid‐containing nanomedicines^[^ ^200^ ^]^ because of their physiological, anatomical, and genetic similarity to humans.^[^ ^201^ ^]^ The United States Food and Drug Administration (FDA) historically mandated toxicity tests for a drug to be conducted on one rodent species (e.g., mice and rats) and one non‐rodent species (e.g., monkeys and dogs) before granting approval for human use, although this requirement is no longer in place as of December 2022 if non‐animal alternative methods can demonstrate safety and efficacy.^[^ ^202^ ^]^ Previously, Stegh and coworkers reported the i.v. injection of gold‐cored SNAs with a 3D shell of siRNA against the oncogene Bcl2L12 for crossing the BBB and treating glioblastoma in mice.^[^ ^116^ ^]^ In 2021, the authors showed that a single i.v. dose of the same SNAs (labeled as NU‐0129) to healthy monkeys did not change body weight, hematology (coagulation and serum chemistry), and cardiovascular function (heart rate, pulse pressure, and electrocardiography) after 14 days of recovery.^[^ ^203^ ^]^ Yet, they detected purple or blue discoloration (or chrysiasis) on body surfaces (e.g., facial area and oral cavity) and in internal tissues (e.g., spleen, liver, gastrointestinal, and urogenital tissues), evidence of systemic deposition of gold. Concerns over gold‐based therapies, such as long‐term accumulation and side effects,^[^ ^204^ ^]^ should inspire future clinical tests of SNAs with biodegradable cores (e.g., liposome and micelle).

### 4.4 Human

**Figure 11:**
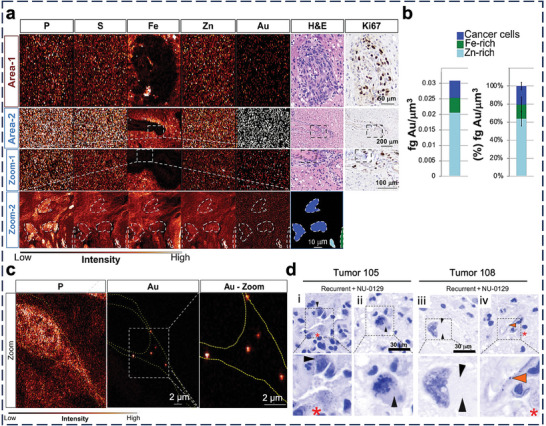
In vivo cell‐NP interaction of SNAs in the glioblastoma tissue of patients. a) Phosphorus (P), sulfur (S), iron (Fe), zinc (Zn), and gold (Au) elemental maps of glioblastoma tumor sample 106. b) Quantification of Au content in Zn‐rich areas, Ki67 (a marker of proliferating cancer cells)‐positive glioma cells, and Fe‐rich areas (left) and cumulative quantification of Au distribution in the different cell populations across different sections and patient tumors (right). c) XFM Bionanoprobe image of a cancer cell demonstrates extranuclear/cytoplasmic localization of Au signal. d) Silver staining of tumor sections post‐NU‐0129 treatment. Au was present within tumor cells (i to iii; black arrowheads), macrophages (i, red asterisks), and ECs (iv, orange arrowhead). Reproduced with permission.^[^ ^203^ ^]^ Copyright 2021, American Association for the Advancement of Science.

Phase 0 clinical trials involve sub‐therapeutic doses of a new drug in several patients or healthy volunteers, with an objective to test if it reaches the diseased site (e.g., tumor) and exerts its intended mechanism of action, before recruitment of larger cohorts.^[^ ^205^ ^]^ The Phase 0 study of NU‐0129 SNAs entailed eight patients with recurrent glioblastoma or gliosarcoma for i.v. infusion at 1/50^th^ of the human equivalent dose based on toxicology data on monkeys.^[^ ^203^ ^]^ ICP‐MS measurements revealed the presence of gold in all tumor specimens, proof of delivery to the tumor. About 41%ID and 81%ID of gold were detected in the tumor for two patients with 159 and 174 d post‐trial enrollment, respectively, proof of long‐term residency. Synchrotron X‐ray fluorescence microscopy (XFM) elemental maps of tumor slices revealed gold accumulation in the zinc/iron‐rich vasculature and inside perivascular and intraparenchymal Ki67‐positive glioma cells (**Figure** **11**a), with ≈20% of total gold found in cancer cells (Figure 11b). High‐resolution XFM, obtained using the Bionanoprobe^[^ ^206^ ^]^ to focus the X‐ray beam to 80 nm, verified the presence of gold in the cell cytoplasm. Light microscopy with silver enhancement staining showed gold accumulation in the extranuclear areas of tumor cells (Figure 11c), within tumor‐resident macrophages, and inside tumor‐associated ECs (Figure 10d). Also, NU‐0129 inhibited Bcl2L12 and upregulated active caspase‐3 and p53. The authors observed only two severe adverse events (AE) (>grade 3; hypophosphatemia and lymphopenia) without grade 4 and 5 AEs through the 21‐day monitoring period; skin discoloration observed in monkeys was not observed in humans post‐monitoring. Gold‐cored SNAs are amenable to various tracking tools to dissect cell‐NP interactions in humans, offering clinical validation of data from mice.^[^ ^116^ ^]^ Still, the long‐term accumulation of gold warrants future studies on skin discoloration in humans.

Phase 1 studies aim to identify the highest dose of a new drug for delivery to healthy volunteers without causing severe side effects. In 2022, Daniel et al. reported a first‐in‐human Phase 1 study to establish the safety and tolerability of delivering single ascending doses of Cavrotolimod to 16 healthy participants, over four dose levels.^[^ ^207^ ^]^ Rather than a classical gold‐cored SNA, Cavrotolimod is a liposome‐cored SNA with a shell of CpG oligonucleotides as TLR9 agonists for cancer immunotherapy. Common AEs were pyrexia, headache, influenza‐like illness, dizziness, and myalgia following s.c. injection to the abdomen, but no severe AEs or dose‐limiting toxicities were observed. Cavrotolimod elicited a broad Th1‐type immune response in a nearly dose‐proportional manner, as evidenced by higher blood levels of interferon‐γ, interleukin (IL)−12 p40, IL‐1RA, IL‐6, IL‐10, and monocyte chemoattractant protein (MCP‐1) that mostly peaked at ≈24 h post‐injection. Lymphocyte activation was detected in NK and T cells, but not B cells, macrophages, or plasmacytoid dendritic cells.

## 5 Discussion and Outlook

Over the past five years, reviews on nucleic acid nanostructures have extensively discussed the issues of in vivo stability,^[^ ^208^, ^209^, ^210^, ^211^ ^]^ safety and immunogenicity,^[^ ^33^, ^208^, ^210^, ^212^, ^213^ ^]^ endosomal escape,^[^ ^208^, ^211^ ^]^ ADME (absorption, distribution, metabolism, and excretion),^[^ ^33^, ^212^, ^213^ ^]^ large‐scale manufacturing and quality control,^[^ ^33^, ^208^, ^212^ ^]^ and structural modification.^[^ ^209^, ^210^, ^212^ ^]^ This review provides complementary future perspectives from the materials and biology angles. To enhance the translational potential of nucleic acid nanostructures and nanomedicines in general,^[^ ^214^ ^]^ it is important to consider both materials design [i.e., delivery of emerging biomacromolecules (Section 5.1) and prediction of nanostructures (Section 5.2)] and biological complexity [i.e., biological tools for probing cell‐NP interactions (Section 5.3) and insights into cellular processes (Section 5.4)]. In Section 5.5, we conclude with some regulatory updates on clinical testing.

### 5.1 Delivery of Biomacromolecules

The global mass deployment of mRNA‐loaded LNP‐based vaccines during the COVID‐19 pandemic is a groundbreaking achievement in nanomedicine, culminating in a Nobel Prize in Physiology or Medicine awarded in 2023. These LNPs often use ionizable lipids to mitigate the side effects associated with the cytotoxicity associated with the positive surface charge of classical liposomes or LNPs. Other cationic NPs for delivering mRNA, such as PEG‐coated polymeric micelles,^[^ ^215^ ^]^ are also on the horizon. Nucleic acid nanostructures should be suitable carriers of mRNA (or other genes^[^ ^216^ ^]^ and large macromolecules) because their negative charge renders them less cytotoxic than cationic NPs,^[^ ^217^ ^]^ but previous studies focused on delivering small molecules and oligonucleotides. It is helpful to expand the experimental toolkit of nucleic acid nanostructures for delivering larger biomolecules. mRNA delivery is more advantageous than plasmid DNA delivery because mRNA does not require nuclear transport for gene expression, yet its delivery to the cytosol is challenging given its length and fragility. Yoshinaga et al. used RNA oligonucleotide staple strands as crosslinking linkers to compact a single‐stranded mRNA to form a <100‐nm NP via hybridization, thus enhancing resistance against RNase by ≈100‐fold.^[^ ^218^ ^]^ Intraventricular injection of the RNA NP into the mouse brain enabled the expression of a reporter gene. The authors later conjugated PEG strands to the exterior of the bundled RNA NP for added stability against serum, but excess PEG attachment reduced cellular entry.^[^ ^219^ ^]^ Hu et al. designed two circular staple RNAs with multiple intermittently spaced binding sites that recognize different segments of mRNA expressing Smad4, a tumor‐suppressing gene; the two circular RNA strands and single‐stranded mRNA self‐assemble to form a 3D lantern‐shaped nanostructure. I.p. injection of the mRNA nano‐lantern reduced tumor growth in mice.^[^ ^220^ ^]^ Tockary et al. constructed a comb‐like mRNA by hybridizing an antigen‐encoding mRNA with a 24‐base pair immunostimulatory short dsRNA (teeth), with its immunostimulation intensity tunable based on the number of teeth. The authors achieved effective cancer vaccination in the mouse lymphoma model with the aid of liposome, LNP, and polymeric micelle transfection. We envision that this modification may benefit the design of nucleic acid nanostructures with simple and compact mRNA strands.^[^ ^221^ ^]^

The Clustered Regularly Interspaced Short Palindromic Repeats (CRISPR)/Cas9 gene editing tool allows for precise edits of the genetic code, but transfection of both the Cas9 protein and single‐guide RNA (sgRNA) is inefficient. Ding et al. designed a DNA‐grafted PCL brush whose DNA sequence is complementary to a segment of sgRNA that does not interact with Cas9.^[^ ^222^ ^]^ After adding the sgRNA/Cas9 ribonucleoprotein complex to the DNA‐polymer brush for self‐assembly, the resultant ≈80‐nm nanogel entered cells and inhibited the target gene in vitro. Tang et al. introduced protospacer adjacent motif (PAM)‐rich regions to a 2D rectangular DNA origami for immobilizing sgRNA/Cas9 complexes at defined regions, followed by rolling it up with lock DNA strands to form a 3D nanotube. I.v. injection into tumor‐bearing mice led to editing of the PLK1 gene and reduced tumor burden.^[^ ^223^ ^]^

These encouraging reports showcase the delivery and efficacy of novel nucleic acid nanostructures for carrying biomacromolecules. Moving forward, it will be instructive to investigate their cell‐NP interactions in vivo in detail.

### 5.2 Prediction of New Nanostructures

Machine learning (ML), a subset of artificial intelligence,^[^ ^224^ ^]^ is an emerging tool for designing nucleic acid nanostructures by employing neural network algorithms trained on large datasets to make predictions.^[^ ^225^ ^]^ Benson et al. ran >500 molecular simulations of a DNA origami nanostructure with >4000 random structural mutations to calculate their effects on structural rigidity in silico, with the beneficial mutations introduced to the next cycle of evolution. Using this dataset to train a neural network, the authors predicted mutation sites that improved local structural rigidity.^[^ ^226^ ^]^ Kim et al. developed a deep‐learning‐based algorithm using high‐ and low‐resolution atomic force microscopy (AFM) images of only one type of origami nanostructure.^[^ ^227^ ^]^ They applied this algorithm to predict the high‐resolution image of several other nanostructure types using their respective low‐resolution AFM images, thus reducing the time of image acquisition and structural characterization. Lee et al. trained an ML model based on neural networks to generate electron densities of a DNA duplex as output using base pairing and base stacking interactions as input. They predicted the electron densities of larger tile‐based junctions and origami nanostructures that were too computationally expensive using conventional quantum methods.^[^ ^228^ ^]^

We envision that the in vivo cell‐NP interactions (e.g., organ‐level distribution, blood pharmacokinetics,^[^ ^229^ ^]^ and cytotoxicity^[^ ^230^ ^]^) of nucleic acid nanostructures can be more accurately predicted via ML; for now, most predictions are in vitro. Yamankurt et al. used high‐throughput screening to measure TLR9 immune activation in macrophages upon incubation with SNAs of ≈1000 different formulations obtained by varying 11 structural parameters (e.g., NP core and antigen location). The authors concluded oligonucleotide chemical modification as a critical parameter, but the effects of many parameters were nonlinear. Using supervised ML to derive structure‐activity relationships based on only ≈16% of the dataset, they accurately predicted TLR activation by the remaining, unselected SNAs.^[^ ^231^ ^]^ For more powerful predictions, we call on the community to rigorously investigate in vivo cell‐NP interactions, thereby generating a sufficiently large and comprehensive dataset for ML training.

### 5.3 Biological Tools for Probing In Vivo Interactions

Meaningful measurements of in vivo interactions are predicated upon choosing animal models that capture the etiology of human diseases. For instance, atherosclerosis in humans predominantly affects the coronary, carotid, and peripheral arteries, whereas most rodents develop plaques in the aorta and its branches. Moreover, human plaques contain more inflammatory cells and lipids but less fibrosis and calcification than animal plaques.^[^ ^232^ ^]^ The complexity and diversity of human atherosclerosis cannot be fully reproduced by any single animal model, therefore different models are needed to address different aspects of the disease.^[^ ^233^ ^]^ In vivo cell‐NP interactions also depend on the disease stage as disease progression may alter the physiology and anatomy of a diseased site, presenting different biological barriers for delivery. For atherosclerosis, blockage of the blood vessel by plaques at different disease stages can alter the blood flow velocity and shear stress^[^ ^234^ ^]^ that may affect cellular uptake and extravasation of NPs.^[^ ^235^ ^]^ For cancer, the delivery, penetration, retention, payload release, and clearance of NPs depend on the tumor microenvironment (e.g., acidity,^[^ ^236^ ^]^ hypoxia,^[^ ^237^ ^]^ immune response^[^ ^238^ ^]^), vascularization, stroma, and macrophage population.^[^ ^239^ ^]^ For kidney fibrosis, we showed that glomerular filtration of NPs to tubules depends on NP size and disease stage,^[^ ^29^ ^]^ noting that deposition of extracellular matrix in the peritubular region affects glomerular hemodynamics and permeability.^[^ ^240^ ^]^ We encourage researchers to consider the effect of disease stage when designing nucleic acid nanostructures for efficacy evaluation.

Two common methods for quantifying cellular‐level distribution in small animals are flow cytometry and fluorescence imaging, both necessitating the use of specific antibodies to sort different cell types in the same diseased organ or tissue. Despite the plethora of rodent‐reactive antibodies for various cell‐specific markers to distinguish among different cell types (e.g., immune cells vs neurons and endothelial vs epithelial), similar antibodies reactive to large animals (rabbits, pigs, and monkeys) are not often commercially available. Moreover, both methods require the fluorescence labeling of the nucleic acid nanostructure and the cell types involved, but they suffer from low sensitivity, and dye‐induced staining artifacts, and cannot inform the in vivo disassembly of the nanostructure into dye or nucleic acid fragments. Wang et al. reported a DNA probe called origamiFISH for detecting DNA origami nanostructures in cells in vitro and tissues ex vivo. After using one set of probe strands to bind to some conserved sequences of the M13mp18 scaffold plasmid, adding another set of fluorescent probe strands initiates a hybridization chain reaction (HCR) to amplify the fluorescence by 1000 folds for detection. Coupling with tissue immunofluorescence and tissue clearing should enable the mapping of DNA nanostructures in different cell types in vivo.^[^ ^241^ ^]^ Similarly, Chen et al. designed an HCR probe for phenotyping diverse immune cell types (CD4^+^, CD8^+^, T cell, and monocyte) in blood samples by conjugating truncated cell‐specific aptamers to a DNA tetrahedron scaffold. The precise spatial orientation and distance control of the two HCR probe strands on the scaffold improved detection sensitivity.^[^ ^242^ ^]^

### 5.4 New Insights into Cellular Processes

Rigorous fundamental studies on cell‐NP interactions will yield new insights into the cellular, organellar, and even molecular processes of nucleic acid nanostructures, ultimately improving the design of nanostructures for delivery or other applications.

Mechanostransduction is a biological process by which cells sense physical forces and generate biochemical signals in response, e.g., proliferation, migration,^[^ ^243^ ^]^ differentiation,^[^ ^244^ ^]^ and apoptosis.^[^ ^245^ ^]^ We studied the interplay between intracellular trafficking and mechanotransduction using SNAs. Surprisingly, compression of cells by a single glass coverslip (only ≈2.2 Pa) for several hours in vitro sufficed to activate the Rho‐associated protein kinase (ROCK) pathway and enhance ROCK‐mediated delivery of SNAs to the cell nucleus (up to 50% accumulation), without inducing cytotoxicity or requiring the use of nuclear localization signals.^[^ ^246^ ^]^ Myosin motors and the nuclear adaptor importin β also mediated intranuclear delivery. Thus, activation of cellular signaling pathways enables the redirection of nucleic acid nanostructures from the endolysosomal pathway to the nucleus. Still, translating to in vivo delivery will require the re‐design of the compression method and pressure site involved.

Without mechanical stimuli, one may simply tune the shape of the nucleic acid nanostructure to modulate cell‐NP interactions. Huang et al. prepared gold NPs with different levels of spikiness modified with DNA oligonucleotides, yielding a spiky version of SNA. NP spikiness induced the recruitment of cellular myosin IIA to the cell membrane and facilitated myosin‐mediated, spikiness‐correlated cellular entry in vitro.^[^ ^247^ ^]^ How this NP performs in vivo, especially when adsorption of blood serum proteins to the NP may change its spikiness and recruitment of myosin, remains to be seen. Dai et al. compacted a ssRNA into 2D RNA origami nanostructures of different edge lengths, thereby giving rise to dsRNA segments. The authors observed a positive correlation between NP edge length and TLR3‐activated immune response in vitro, noting that dsRNA is a ligand of TLR3.^[^ ^248^ ^]^ Intratumoral injection of self‐therapeutic RNA origami nanostructures elicited an anti‐tumor immune response in mice.

One may leverage the spatial addressability of nucleic acid nanostructures by loading biomolecules at defined locations relative to cell‐surface receptors for activating cellular responses. Wang et al. patterned multiple tumor necrosis factor (TNF)‐related apoptosis‐introducing ligand (TRAIL)‐mimicking peptides, a ligand of death receptors 4 and 5 on cancer cells, in the form of a hexagonal array with varying interpeptide spacing on a 2D DNA origami nanostructure.^[^ ^249^ ^]^ The authors identified 5 nm as the most suitable interpeptide distance for inducing death receptor clustering and apoptosis, offering insights into NP‐receptor interaction. They envisioned DNA nanostructures as a platform for drug screening in vitro, but it will be interesting to check if they cluster the same receptors and induce apoptosis in vivo.

### 5.5 Regulatory Updates

In 2022, the United States FDA finalized its guidance on NP‐containing drug products, including biological.^[^ ^250^ ^]^ When compared to free drugs, the guidance calls for more detailed information on the interactions of NP‐based drugs with the biological system, including the exposure, responses, the role of enzymes, and immunogenicity, all factors in line with our emphasis on in vivo cellular response. When evaluating safety risks, the guidance emphasizes adequate i) characterization of NPs and ii) understanding of how NP attribute relates to quality, safety, and efficacy. Some recommended factors for assessment are how NP attributes impact in vivo pharmacokinetics, drug release and degradation, bioavailability, and distribution, consistent with our call for understanding in vivo cellular‐level distribution.

Different countries and regions (e.g., the United States, Europe, Brazil, Japan, South Korea, and China) have established their own regulatory frameworks over the human tests of “advanced therapy products (ATPs)” over the past decade.^[^ ^251^ ^]^ Broadly, ATPs encompass gene therapy, cell engineering, and tissue engineering products. Nucleic acid nanostructures for delivering gene cargoes fall under the category of gene therapy. Regulations over gene therapies mostly oversee the risks of random gene insertion, immune system activation, and off‐targeting. As an example, in Hong Kong, the regulatory framework for ATPs came into operation in 2021, and the ATP Good Manufacturing Practice Center (inaugurated by The Chinese University of Hong Kong and Hong Kong Science and Technology Parks Corporation) to produce clinical grade ATPs for trials and treatments formally opened in 2023. Such exciting developments will foster the clinical translation of nucleic acid nanotechnology.

## Conflict of Interest

The authors declare no conflict of interest.
